# BioVector, a flexible system for gene specific-expression in plants

**DOI:** 10.1186/1471-2229-13-198

**Published:** 2013-12-05

**Authors:** Xu Wang, Chengming Fan, Xiaomei Zhang, Jinlong Zhu, Yong-Fu Fu

**Affiliations:** 1 MOA Key Lab of Soybean Biology (Beijing), National Key Facility of Crop Gene Resource and Genetic Improvement, Institute of Crop Sciences, Chinese Academy of Agricultural Sciences, 12 Zhongguancun Nandajie, Haidian District, Beijing 100081, China

**Keywords:** Expression vector, Gene specific-expression, Functional genome study, Gene cloning, Restriction enzyme/ligase strategy, Gateway DNA recombination

## Abstract

**Background:**

Functional genomic research always needs to assemble different DNA fragments into a binary vector, so as to express genes with different tags from various promoters with different levels. The cloning systems available bear similar disadvantages, such as promoters/tags are fixed on a binary vector, which is generally with low cloning efficiency and limited for cloning sites if a novel promoter/tag is in need. Therefore, it is difficult both to assemble a gene and a promoter together and to modify the vectors in hand. Another disadvantage is that a long spacer from recombination sites, which may be detrimental to the protein function, exists between a gene and a tag. Multiple GATEWAY system only resolves former problem at the expense of very low efficiency and expensive for multiple LR reaction.

**Results:**

To improve efficiency and flexibility for constructing expression vectors, we developed a platform, BioVector, by combining classical restriction enzyme/ligase strategy with modern Gateway DNA recombination system. This system included a series of vectors for gene cloning, promoter cloning, and binary vector construction to meet various needs for plant functional genomic study.

**Conclusion:**

This BioVector platform makes it easy to construct any vectors to express a target gene from a specific promoter with desired intensity, and it is also waiting to be freely modified by researchers themselves for ongoing demands. This idea can also be transferred to the different fields including animal or yeast study.

## Background

Along with the fulfilment of the whole genome sequencing of different organisms including many crops (http://genomesonline.org/cgi-bin/GOLD/index.cgi?page_requested=Statistics), the challenge of how to comprehensively study gene functions on a large scale emerges. Gene cloning, gene expression in different modes (such as constitutive, tissue-/developmental-specific, or inducible ectopic expression), protein localization and interaction, silencing the gene, and promoter function and regulation are always the issues a researcher concerns. Therefore, a series of high efficient cloning and expression vectors are developed in different labs [1-10]. The primary cloning approaches available can be mainly classed into two groups: the traditional restriction enzyme/ligase (REL) strategy including TA cloning technology and the DNA recombinational cloning systems including the Gateway cloning system from Invitrogen and the Creator cloning system from CLONTECH. The former restriction enzyme/ligase strategy, even though cheap, suffers from various problems, mainly no suitable restriction enzyme sites in binary vectors compatible for different genes and promoters. Therefore, it is rather limited for projects on a large scale and multiple functions. TA cloning technology increases the cloning efficiency, and at the same time it increases the cost too, because it is difficult to generate a high-quality TA cloning vector in home-made and a researcher has to purchase different TA cloning kits from the market.

The DNA recombinational cloning system, especially the Gateway Cloning System from Invitrogen, is recently developed and widely used. The first step of this system (BP recombination reaction) is to clone the PCR product into a donor vector to produce an entry clone [11]. Once an entry clone is available, the gene of interest is easily transferred into different expression vectors through an LR reaction, which is a high efficient reaction. Due to its many advantages, many cloning systems related were developed [1-4]. However, there are still some limitations for this technology. For example, the primers at the first step need a long extra sequence for recombination reaction, which decreases the efficiency of PCR and BP reaction and increases the cost. Aimed at these problems, higher efficiency vector systems are developed. Invitrogen develops a high efficiency kit (TOPO Gateway Entry vectors), which combines TA cloning and BR reaction to eliminate the BP reaction, and the resulting vector can be directly used to LR reaction (http://www.invitrogen.com/), but it is at the cost of a high price. The ZeBaTA system introduces the TA cloning into plant binary vectors and results in zero background vectors [12]. This ZeBaTA system obviously needs to make a lot of home-made and low efficiency T-binary-vectors for different purposes, such as to label a gene of interest with different tags. TA cloning has another problem waiting to be solved: the gene orientation, which decreases the cloning efficiency. Multiple Gateway Systems are developed for cloning multiple DNA fragments (such as, promoters, genes and terminators) into a expression vector in one step [5-8], but it also suffers from low efficiency because of many recombination sites. The type IIs endonucleases recognize asymmetric DNA sequences, 4–7 bp long, and cleave both strands at specific locations up to 20 bases away from their recognition site [13]. Recently, two excellent papers reported new gene cloning strategies, referred to Golden Gate Cloning (mainly based on the type IIs endonucleases *Bsa* I and *Bbs* I) [9] and GoldenBraid (mainly based on the type IIs endonucleases *Bsa* I and *Bsm*B I) [10], with which it is possible to seamlessly assemble multiple reusable gene modules, including promoters, genes and terminators, together in a binary vector in a single restriction-ligation.

Even though a lot of cloning systems are now available as discussed above, many common problems still exist in our routine experiment. In modern functional genomic study, one of challenges is to construct a set of vectors for a gene of interest to study gene functions in special spatio-temporal patterns. It includes cloning rapidly and efficiently, labeling proteins with different tags, shuffling randomly promoters and genes, introducing a desired regulatory element for a given promoter or gene, and modifying vectors in hand for ongoing demands. Therefore, the flexibility of vectors appears to be a main challenge. What’s more, GATEWAY technology available now leaves a common question to be answered: after LR reaction, there is a longer spacer existed between genes and tags, which may result in detrimental effect on protein functions [14].

In this study, we developed a highly flexible expression system to cope with on-going demands in plant functional genomic study, based on classical cloning approaches other than to introduce a new method. Firstly, we employed widely-used site-specific recombination (SSR) cloning system (Gateway, Invitrogen) for high cloning efficiency. Secondly, we introduced two pairs of SSRs, one for genes and the other for promoters in two independent entry clones (EC). Thirdly, we adopted traditional restriction enzyme/ligase (REL) strategy in an easy-operating intermediate cloning vector, instead of a difficult-operating binary vector as previous researchers used. This REL is designed for cloning of desired multiple regulatory elements or tags, and it also provide flexible choices for cloning.

## Results and discussion

Through directly synthetizing sequences of all DNA fragments (Additional file 1) except the backbone of vectors indicated below, we developed our cloning system, BioVector. BioVector is composed of three basic vectors: a gene entry clone (GEC) with one pair of SSRs of *att*L1/*att*L2, a promoter entry clone (PEC) with another pair of SSRs of *att*L3/*att*L4, and a binary destination vector (BDV) with two pairs of corresponding SSRs of *att*R1/*att*R2 and *att*R3/*att*R4. Therefore, with a single LR reaction (Invitrogen), a promoter can be fused to a gene and a plant expression vector is constructed.

### Gene entry clone

**Figure 1:**
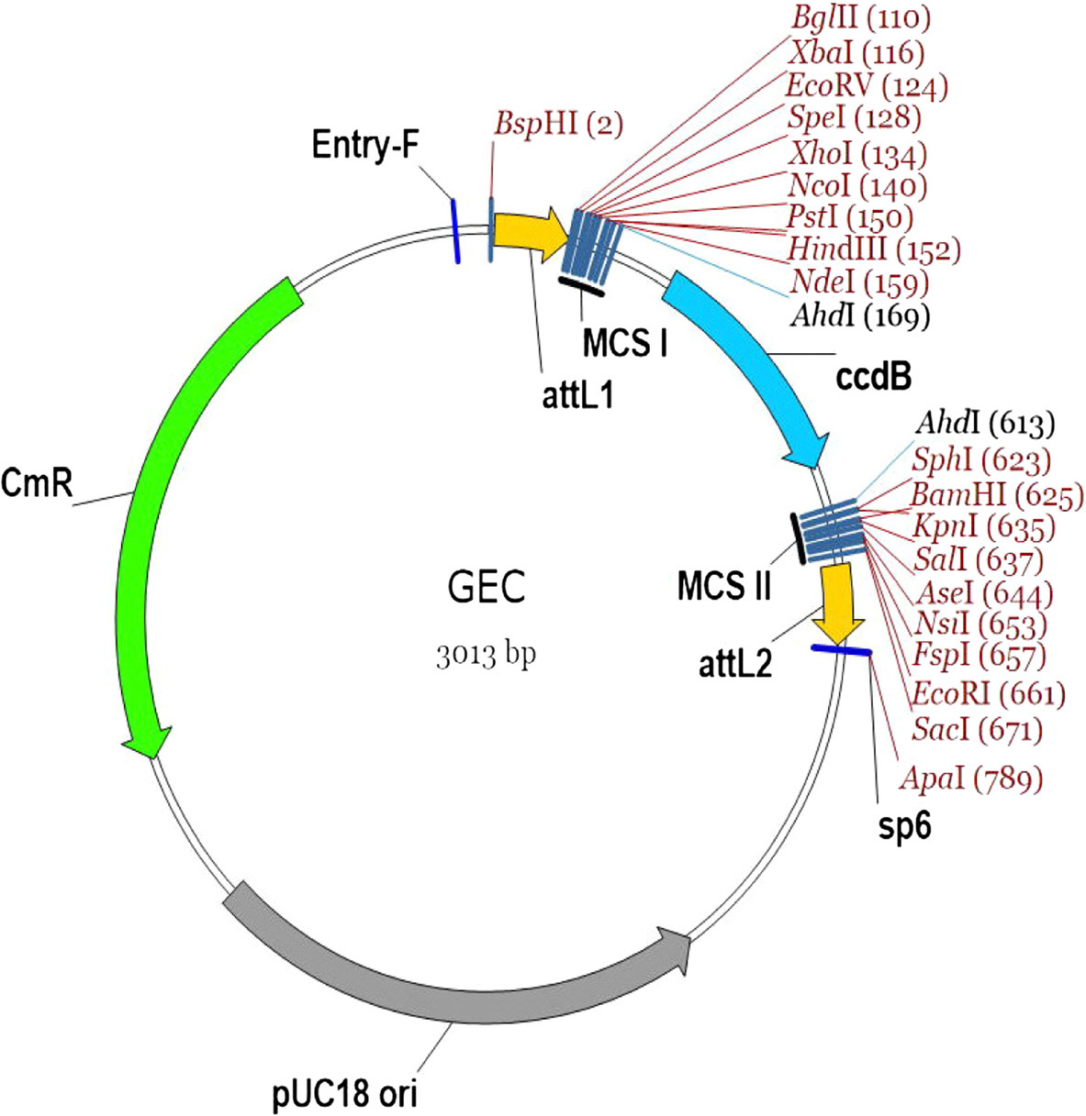
**The general map of GECs.** MCS I and MCS II, the multiple cloning sites; *att*L1 and *att*L2, recombination sites; *ccd*B, a negative selection marker for DH5α; CmR, Chloramphenicol resistant in *E. coli*; Entr-F and sp6, sequencing primers; the numbers in parenthesis, the position of corresponding restrict enzymes.

The gene entry clone (GEC) (Figure 1, Table 1) was developed from the pUC vector due to its advantage of easy-operating for cloning, and introduced up to 20 restriction enzyme/ligase (REL) sites including T/A cloning sites (*Ahd* I sites), therefore it is convenient to clone any DNA fragments and to assemble multiple DNA fragments into a GEC. REL sites were inserted inside site-specific recombination (SSR) sites (*att*L1 and *att*L2) in GECs, so that it was possible to make a seamless fusion between a gene and a tag in our system, rather than to introduce a SSR spacer (around 20 aa) between them as widely-used GATEWAY Cloning System does, in which the tags are in destination vectors [1,7,15]. Such a spacer has the potential to result in the synthesis of a non-functional or insoluble protein-tag fusion [14]. Another improvement was that a stop code was put at the end of REL sites and inside SSR (*att*L2). Thus, only the gene without a stop code was needed to be cloned into a GEC, then it can be removed from one GEC to another by a simple REL. In this case, there was no need to clone a gene for the N- or C-terminal fusion, respectively, as wide-used GATEWAY Cloning Systems do [1-4]. Therefore, GECs saved both time and cost for cloning and sequencing of genes.

**Table 1:** **A list of gene entry clones** (**GEC**)

**Vector name**	**Tag and its position**	**Restriction enzyme sites at both ends of tags**	**ABRC stock number**✶
Fu28	C-GFP	*Sac* I	CD3-1822
Fu30	N-GFP	*Bgl* II	CD3-1823
Fu41	N-YFP	*Bgl* II, *Xba* I	CD3-1824
Fu42	C-YFP	*Eco*R I, *Sac* I	CD3-1825
Fu43	N-CFP	*Bgl* II, *Xba* I	CD3-1826
Fu44	C-CFP	*Eco*R I, *Sac* I	CD3-1827
Fu45	N-mRFP	*Bgl* II, *Xba* I	CD3-1828
Fu46	C-mRFP	*Eco*R I, *Sac* I	CD3-1829
Fu47	N-3MYC	*Bgl* II	CD3-1830
Fu48	C-3MYC	*Sac* I	CD3-1831
Fu49	N-3FLAG	*Bgl* II	CD3-1832
Fu50	C-3FLAG	*Sac* I	CD3-1833
Fu55	N-3HA	*Bgl* II	CD3-1834
Fu56	C-3HA	*Eco*R I	CD3-1835
Fu58	N-GST	*Bgl* II, *Xba* I	CD3-1836
Fu59	N-StrepII	*Bgl* II	CD3-1837
Fu60	N-NLS	*Bgl* II	CD3-1838
Fu61	C-NES	*Sac* I	CD3-1839
Fu62	C-GUS	*Eco*R I, *Sac* I	CD3-1840
Fu63	C-LUC	*Eco*R I, S*a*c I	CD3-1841
Fu64	N-FLAG; C-YFPc	*Bgl* II for FLAG;	CD3-1842
		*Eco*R I, *Sac* I for YFPc	
Fu65	N-MYC; C-YFPn	*Bgl* II for MYC;	CD3-1843
		*Eco*R I, *Sac* I for YFPn	
Fu66	N-YFPc; C-HA	*Bgl* II, *Xba* I for YFPc;	CD3-1844
		*Sac* I for HA	
Fu67	N-YFPn; C-MYC	*Bgl* II, *Xba* I for YFPn; *Sac* I for MYC	CD3-1845
Fu79	Tag free		CD3-1846

✶All vectors were delivered to ABRC (http://www.arabidopsis.org/). “N” or “C” indicates that the position of a tag is at the “N” or “C” terminus of a target gene, “n” or “c” for the “n” or “c” half part of *YFP* fluorescence protein. All vectors contain recombination sites of *att*L1 and *att*L2 and have the chloramphenicol selection marker in *E. coli*. All the sequences are showed in Additional file 1.

Therefore, genes, tags, and any regulatory elements of interest from PCR amplification can be directly cloned into GECs after digestion. We constructed a set of alternative GECs for labeling a desired protein with different tags at the N- or C-terminus (Table 1). And all GECs shared identical REL sites, and it was easy to move a gene from one GEC to another with a simple REL.

A *ccd*B gene in REL sites (Figure 1) served as a negative selection marker for *E. coli* DH5α as all GATEWAY entry vectors employ (Invitrogen) [1,7,15]. The chloramphenicol (Cm) was employed as a selection marker in *E. coli*, which may be compatible to most of binary vectors available.

We designed a primer Entry-F (5′-ACTTGCATTACAGCTTACGAACCGA-3′) for forward sequencing, and the general primer sp6 (5′-ATTTAGGTGACACTATAG-3′) can be used as a reverse sequencing primer.

Plenty of REL sites in GECs provide a flexible platform for *ad arbitrium* modifying the vector by researcher themselves for their own individual study. This is an important respect of this flexible system, because the plasticity of a system is always demanded in functional genome study, but a shortage of wide-used GATEWAY systems [1-4]. For example, to insert RNA-binding loops (16× BoxB or 6× MS2) [16] inside SSRs in GEC makes it possible to label an RNA.

One of our considerations was to develop GECs compatible with the present Gateway destination vectors for plants, *E. coli*, and yeast, so researchers can keep their vectors in hands when introduce BioVector.

### Promoter entry clone

**Figure 2:**
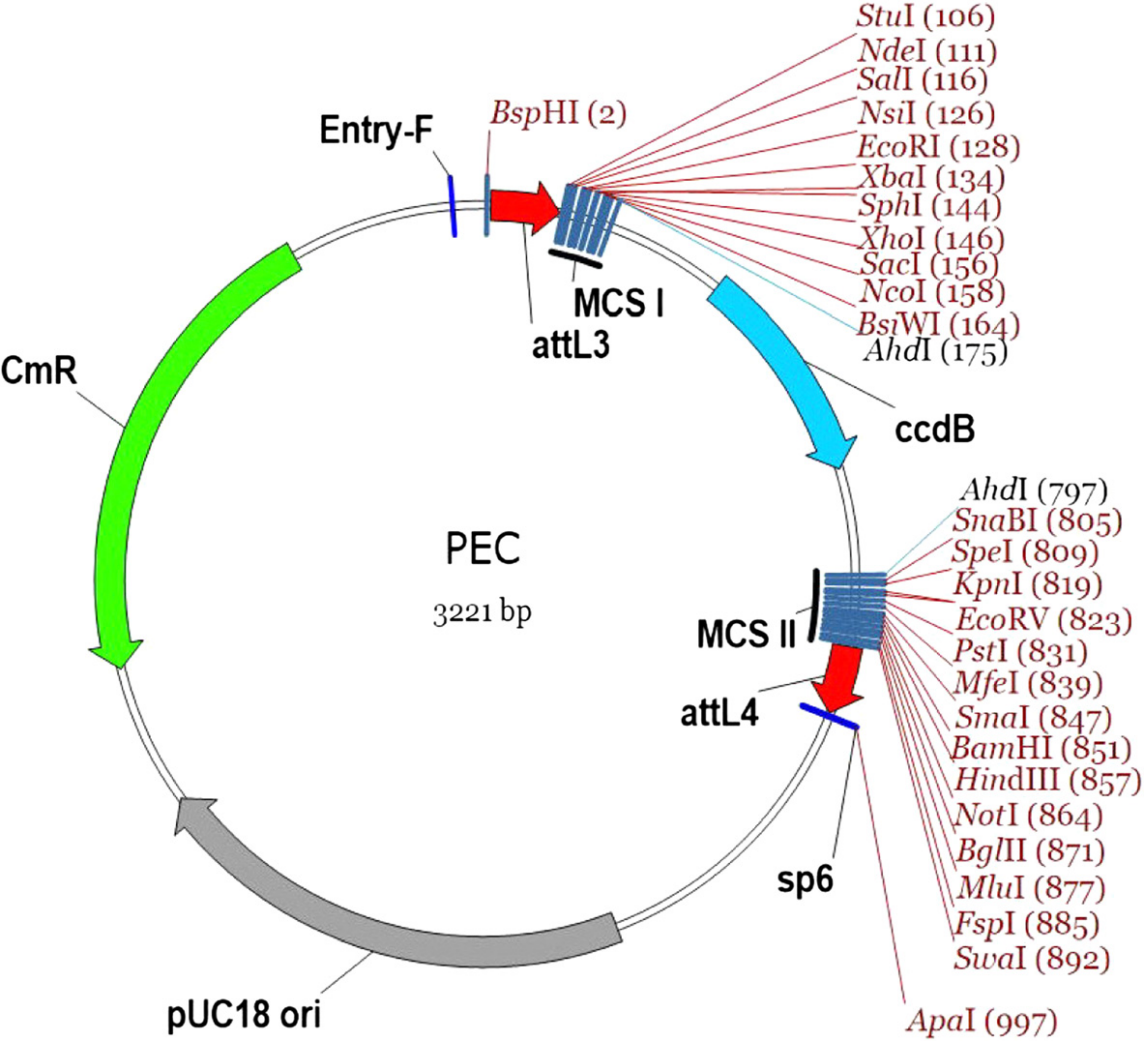
**The general map of PECs.** Notes are same as in Figure 1.

Most of GATEWAY based cloning systems normally put a constitutive promoter, such as CaMV 35S, in the binary vector, in which the promoter is not easy to be replaced by another promoter due to its low cloning efficient. Even though Multiple Gateway System [5-8] clone a promoter inside the specific SSR sites, it bears low efficient in subsequent multiple recombination reactions and it is also difficult to insert a *cis*-element next to the promoter. The promoter entry clone (PEC) in BioVector (Figure 2) also originated from pUC vector, shared the same backbone as GEC, and embraced up to 27 REL sites inside SSR sites (*att*L3 and *att*L4). The plenty of REL sites benefited to freely clone or assemble various promoters or *cis*-elements together to study their functions. Obviously, as in GECs, PECs were good to be updated by researchers for an ambitious idea.

There were three PECs available with no enhancer, one enhancer, or two enhancers, respectively (Table 2). Thus, a gene can be expressed or over-expressed from a native promoter, and the *bona fide* functions of a gene could be revealed. Such a strategy was designed to avoid disadvantages of generally over-expressing promoter CaMV 35S, which has overt ectopic effects [17], weak or null functions in some tissues and plants [18-22], and adverse effects on adjacent genes [19,23,24].

**Table 2:** **A list of promoter entry clones** (**PEC**)

**Vector name**	**Number of enhancers**	**Restriction enzyme sites at both ends of enhancers**	**ABRC stock number**✶
Fu76	Enhancer free		CD3-1847
Fu77	1 × Enhancer	*Nde* I	CD3-1848
Fu78	2 × Enhancer	*Nde* I	CD3-1849

✶ All vectors were delivered to ABRC (http://www.arabidopsis.org/). All vectors contain recombination sites of *att*L3 and *att*L4 and have the chloramphenicol selection marker in *E. coli*. All the sequences are showed in Additional file 1.

Again, the three PECs had identical REL sites, which conferred to easily shift promoters among them. The selection marker and the sequencing primers in PECs were same to that of GECs.

Besides REL strategy, other cloning technology, such as In-Fusion® HD Cloning from Clontech (http://www.clontech.com, Protocol No. PT5162-1, October 2011), can be an alternative efficient approach for cloning genes or promoters into GECs or PECs, so as to increase the cloning efficiency. If introducing the type IIs endonucleases, such as *Bsa* I, *Bbs* I, and *Bsm*B I, GECs and PECs could be compatible to Golden Gate Cloning [9] and GoldenBraid [10].

### Binary destination vector

All binary destination vectors (BDVs) in this study were developed from pGreen/pCLEAN, which are highly efficient in a wide arrange of plants [25,26]. So, BDVs have smaller size (5 ~ 9Kb) than most of gateway-compatible binary vectors (10 ~ 18Kb) [1,3,4]. And the small size of binary vectors facilitates to increase the cloning efficiency, plasmid yield and plant transformation [27]. Because the right border of the T-DNA is largely preserved whereas the left border is frequently truncated after integration [17,28,29], a selectable marker in *planta* was placed next to the left border in BDVs to ensure that all transformants with a positive marker always carry the introduced gene.

**Figure 3:**
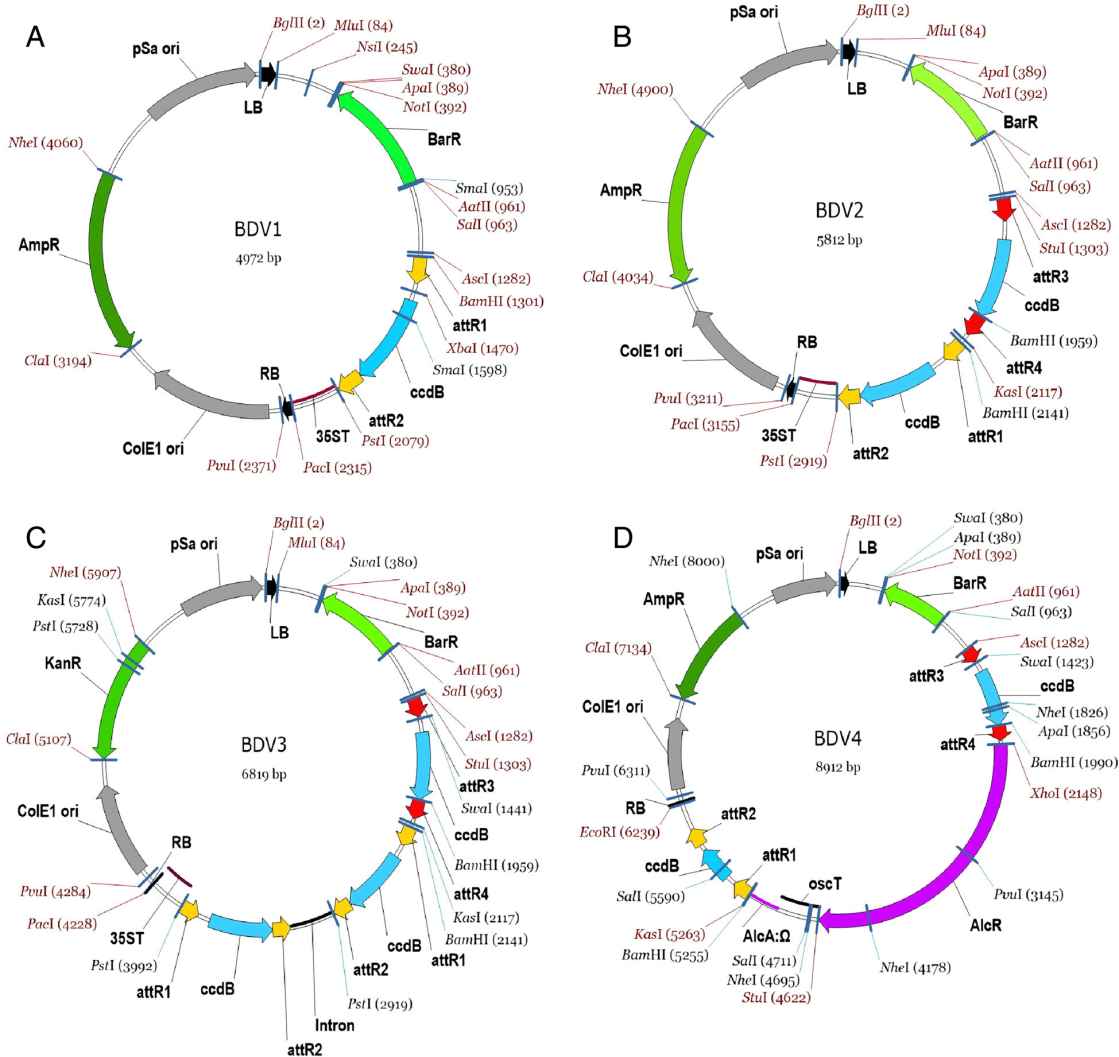
**The maps of four kinds of BDVs. A**, a BDV for expression of genomic gene; **B**, a BDV for expression of coding sequence of a gene from a specific promoter; **C**, a BDV for expression of a gene fragment from specific promoter to silence a gene in specific temporal- or spatio- mode; **D**, a BDV for expression of a gene from both ethanol inducible and specific promoter to fulfill artificially expressing a gene from a native promoter. All the sequences are showed in Additional file 1. *att*R1/2/3/4, recombination sites; LB/RB, the left or right border of T-DNA; *35ST* and *oscT*, terminators; AmpR, KanR, and BarR, selection markers in *E. coli* or plants; *AlcR*/*AlcA*, ethanol promoter. Other notes are same as in Figure 1.

A set of binary vectors were designed for a genomic gene (BDV1, Figure 3A) or CDS gene expression (BDV2, Figure 3B), gene silencing (BDV3, Figure 3C), and ethanol-inducible expression (BDV4, Figure 3D), respectively. There were more BDVs available with various selection markers in *E. coli* and plants (Table 3), providing multiple choices in different projects. So, BioVector can be used to express a genomic gene spanning the sequence from the promoter to the terminator, to analyze the function of coding sequence from a desired promoter or ethanol-inducible promoter, to monitor proteins with fluorescent and other tags, to study protein-protein interaction, and to silence a gene in specific spatio-temporal mode. The MCS in BDVs facilitates to be modified for extensive demands (Figure 3), such as replacing the preloaded transcription terminator or the selection marker.

**Table 3:** **A list of binary destination vectors** (**BDV**)

**Vector names**	**Vector type**	**Selection markers in *E. coli***	**Selection markers in plants**	**Comments**	**ABRC stock number**✶
Fu39-14	BDV1	Ampicilin	Glufosinate	To express a genomic gene	CD3-1850
Fu39-15	BDV1	Kanamycin	Glufosinate		CD3-1851
Fu36-2	BDV1 with 35S promoter	Ampicilin	Glufosinate	To express a gene from 35S promoter	CD3-1852
Fu39-1	BDV2	Ampicilin	Glufosinate	To express a gene from a promoter of interest	CD3-1853
Fu39-2		Kanamycin	Glufosinate		CD3-1854
Fu39-3		Spectinomycin	Glufosinate		CD3-1855
Fu39-4		Ampicilin	Kanamycin		CD3-1856
Fu39-5		Kanamycin	Spectinomycin		CD3-1857
Fu39-6		Kanamycin	GFP		CD3-1858
Fu39-7		Kanamycin	CFP		CD3-1859
Fu39-10		Kanamycin	mRFP		CD3-1860
Fu39-11		Kanamycin	Kanamycin		CD3-1861
Fu39-12		Ampicilin	mRFP		CD3-1862
Fu39-13		Ampicilin	Spectinomycin		CD3-1863
Fu39-9	BDV3	Kanamycin	Glufosinate	To silence a gene	CD3-1864
Fu39-8	BDV4	Ampicilin	Glufosinate	To express a gene from ethanol inducible promoter	CD3-1865

✶All vectors were delivered to ABRC (http://www.arabidopsis.org/). All the sequences are showed in Additional file 1.

**Figure 4:**
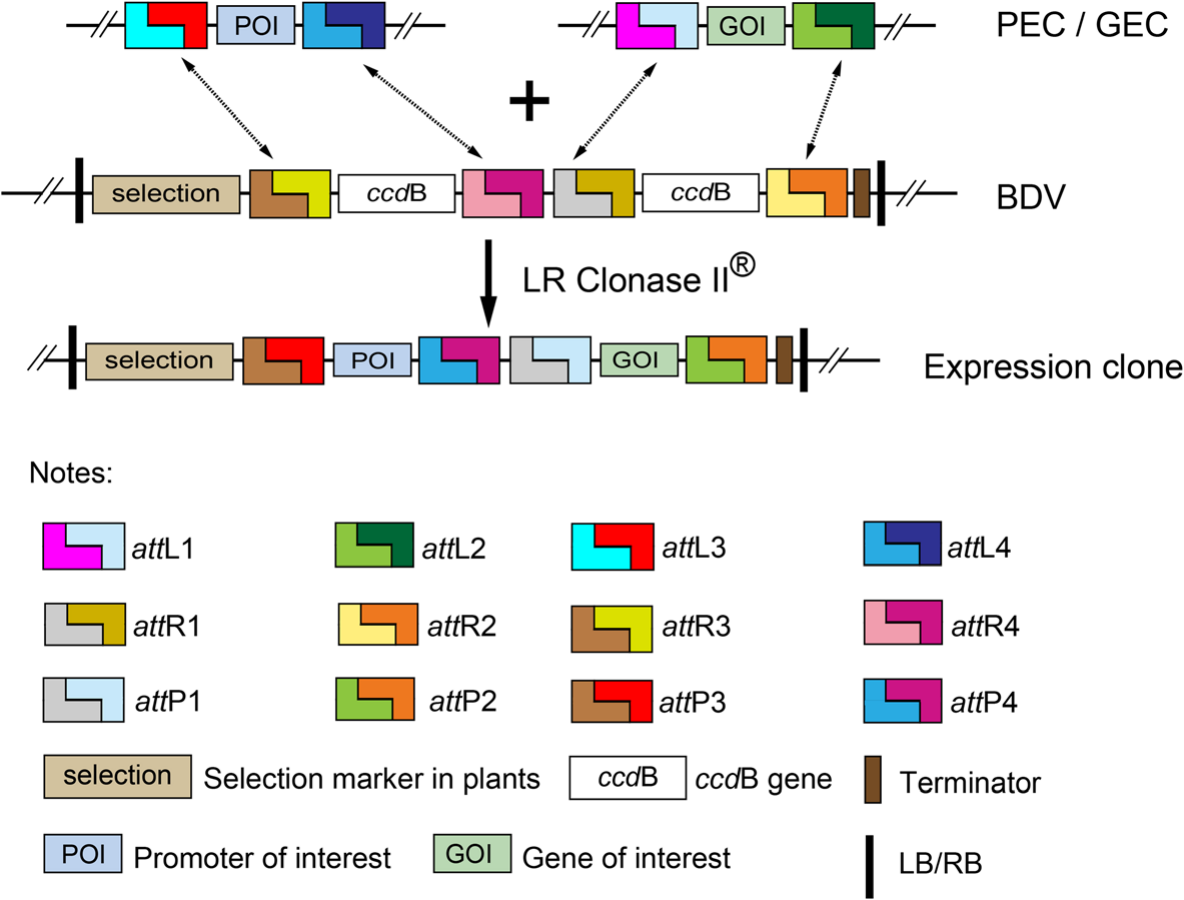
**The rationale of BioVector.** The LR reaction among GEC, PEC and BDV shows the basic principle of BioVector, in which a gene and a promoter in individual entry clones are positioningly and directionally joined together in a destination vector. *att*L1/2/3/4, *att*R1/2/3/4, and *att*P1/2/3/4, recombination sites; *ccd*B, a negative selection marker for DH5α; LR Clonase II®, recombination enzyme from Invitrogen.

Once a gene and a promoter were cloned into GECs or PECs, respectively, the gene and the promoter can be assembled into a binary vector through a single LR reaction (Figure 4), which is a high efficient and directional reaction (Invitrogen). Several systems, such as MultiSite Gateway Cloning [15], provide a possibility to combine promoters and genes at will, but with limited numbers of promoters and tags available. BioVector, however, made it easy to express a gene from different desired promoters with different intensities.

Gene entry clones from wide-used GATEWAY system made by most researchers shared the same SSR sites *att*L1/2 at both ends with GECs. Thus, Gene entry clones at hand were compatible with our BDVs, rather than discarding them when introduce BioVector.

Obviously, GECs and PCEs can serve as an intermediate vector for cloning due to plenty of MCSs available (Figure 1 and 2). In addition, GECs, PECs and BDVs can be shared worldwide as a library. We have delivered all vectors in this study to ABRC centre (Table 1, 2 and 3).

### Verification of BioVector

**Figure 5:**
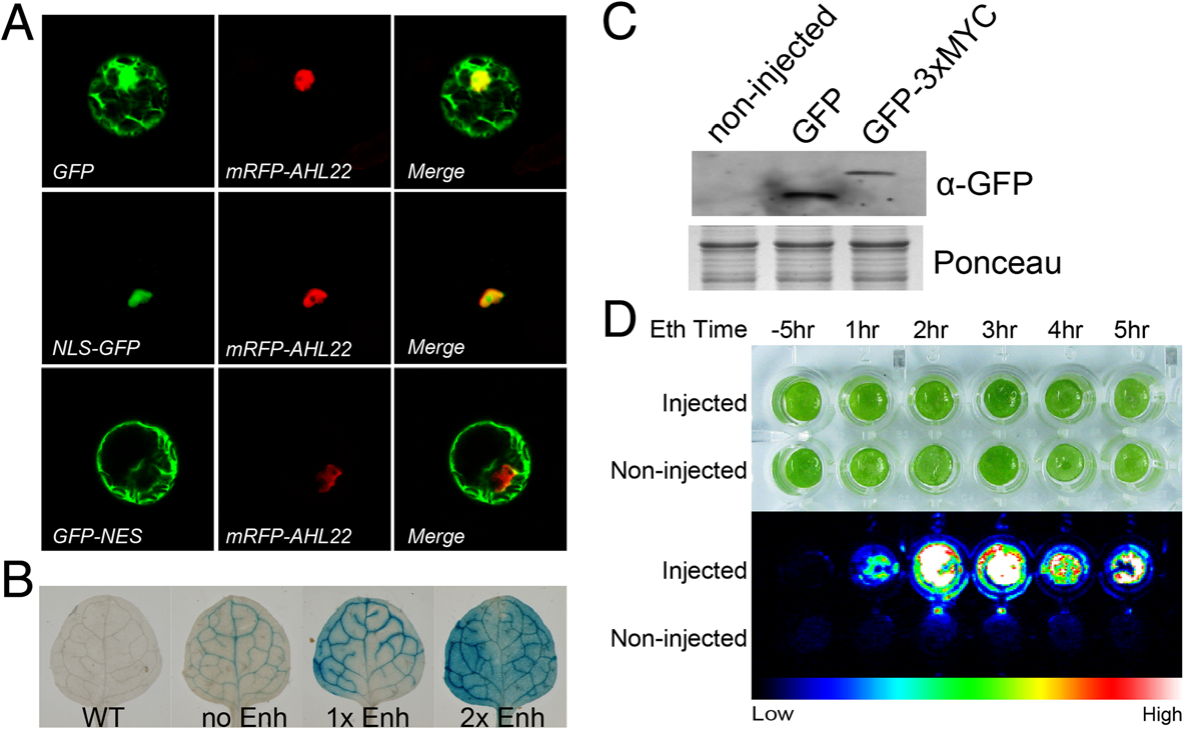
**The verification of BioVector. A**, Analysis of signal peptides. The expression constructs, Fu39-2-*35S*::*NLS*:*GFP*, Fu39-2-*35S*::*GFP*:*NES*, or Fu39-2-*35S*::*GFP*, respectively, were co-transfected with a nuclear protein marker (AHL22, [31]) construct (*35S*::*mRFP*-*AHL22*) into *Arabidopsis* protoplasts, and the fluorescence signal was observed under a confocol microscope after 14 hours incubation. **B**, Analysis of promoter activity. The constructs of Fu39-2-*SUC2*::*GUS*, Fu39-2-*SUC2*:*Enh*::*GUS*, Fu39-2-*SUC2*:*2xEnh*::*GUS* were transformed into *Arabidopsis* (Col). T1 transgenic plants for each construct were analyzed with GUS staining. **C**, Detection of tagged proteins. The Fu39-*2X35S*::*GFP* and Fu39-*2x35S*::*GFP*:*3xMyc* expression constructs were respectively introduced into *Arabidopsis* protoplasts, and the protein was extracted, subjected to SDS-PAGE, and then probed by anti-GFP antibody on a western blot. **D**, Ethanol induced expression of LUC. Fu39-8-*Ub*::*LUC* was infiltrated into 3-week-old *N. benthamiana* leaves mediated by *Agrobacterium*. The leaf disc was harvested on day 3 after infiltration and incubated in 1/2 MS liquid medium containing 2% (v/v) ethanol for indicating hours. Then the luciferin was added into the medium to a final concentration at 100 μM, and the bioluminescence signal was imaged by a CCD camera (Princeton). The bright field and luciferase imaging were respectively shown in the upper and lower panel. All experiments were carried out with at least three biological replicates.

With BioVector we succeeded in stable or transient expression of GFP with or without nuclear localization signal (NLS) or nuclear export signal (NES) in *Arabidopsis* protoplasts (Figure 5A), a *GUS* gene from the companion cell-specific *SUCROSE H*^+^*SYMPORTER2* (*SUC2*) promoter [30] with or without enhancers in *Arabidopsis* (Figure 5B), a Myc-tagged gene in *Nicotiana benthamiana* (Figure 5C), and a luciferase gene under ethanol-inducible pattern *N. benthamiana* (Figure 5D). The results supported that BioVector was efficient expression vector for plants.

## Conclusion

Perfect combination of conventional REL strategy and modern SSR technology confers obvious advantages to BioVector. (1) Exchangeable and Efficient. A gene and a promoter can be easily assembled together to fulfill expressing a gene from a temporal-spatio promoter with different intensity, especially overexpressing genes under the control of native promoters; (2) Flexible. GECs, PECs, and BDVs can be *ad arbitrium* modified with ongoing demands; (3) Practical and Versatile. BioVector can be applied to almost all fields in functional genome research of various plants; (4) Universal and Time-/Labour-Saving. GECs can be efficiently applied to any plant, yeast, and *E. coli* destination vectors sharing corresponding SSR sites, and it is possible to construct a worldwide library as shared community resource for GECs, PECs and BDVs; (5) Seamless fusion. It is possible to make seamless fusion between a protein and a tag, rather than to introduce a detrimental SSR spacer as the widely-used Gateway recombination system does; (6) Broad application and interest. The idea of BioVector can also be applied to similar study in animals and yeast.

## Methods

### Vectors constructing

All basic vectors including gene entry clones, promoter entry clones, and binary destination vectors, were directly synthetized according to the sequences on Additional file 1. Then the PCR amplified products of different tags, fluorescent markers, selection markers, and other elements were inserted into corresponding positions by restriction enzyme/ligase (REL) strategy to produce a set of vectors (Figure 1~3, Table 1~3). We deliverer all vectors in this study to TAIR, so that all vectors can be ordered from TAIR (http://www.arabidopsis.org/).

### Gene and promoter cloning

The original *SUC2* promoter is kindly presented by Dr. George Coupland. For making an entry clone of *SUC2*, the sequence of *SUC2* was cloned into Fu76, Fu77, and Fu78 between *Sal* I and *Bgl* II sites, respectively, to produced Fu76-*SUC2*, Fu77-*SUC2*, and Fu78-*SUC2*. The *Ubiquitin* (*Ub*) promoter was inserted between *Stu* I and *Fsp* I sites in Fu76 to generate Fu76-Ub. *2x35S* promoter were subcloned from pLeela (from Dr. George Coupland) by PCR and inserted into Fu76 with *Sal* I and *Pst* I restriction sites to make Fu76-*2x35S*. Fu62 was digested with *Bgl* II and *Bam*H I to remove excess MCS and self-ligated to produce Fu62-*GUS*. Fu63 was digested with *Bgl* II and *Bam*H I and self-ligated to make Fu63-*LUC*. The *GFP* gene was cloned into Fu26, Fu48, Fu60, and Fu61, respectively, to generate expression vectors of Fu26-*GFP*, Fu48-*GFP*:*3xMyc*, Fu60-*NLS*:*GFP*, and Fu61-*GFP*:*NES*. All genes and promoters were confirmed by sequencing with primers of Entry-F (5′-ACTTGCATTACAGCTTACGAACCGA-3′) and sp6 (5′-ATTTAGGTGACACTATAG-3′).

### LR reaction protocol

Fu26-*GFP*, Fu48-*GFP*:*3xMyc*, Fu60-*NLS*:*GFP*, and Fu61-*GFP*:*NES*, respectively, with Fu76-*2x35S* recombined with Fu39-2 through LR reaction (Invitrogen) to make binary vectors of Fu39-*2x35S*::*GF*P, Fu39-*2X35S*::*GFP*:*3xMyc*, Fu39-*2x35S*::*NLS*:*GFP*, Fu39-*2x35S*::*GFP*:*NES*. And Fu76-*SUC2*, Fu77-*SUC2*, and Fu78-*SUC2*, respectively, with Fu61-*GUS* was subjected to LR reaction (Invitrogen) with Fu39-2 to make *promoter*::*GUS* expression vectors, Fu39-2-*SUC2*::*GUS*, Fu39-2-*SUC2*:*Enh*::*GUS*, Fu39-2-*SUC2*:*2xEnh*::*GUS*. Both Fu63-*LUC* and Fu76-*Ub* were subjected to LR reaction with Fu39-8 to get ethanol-induced *LUC* expression vector Fu39-8-*Ub*::*LUC*. 5 μL reaction system was used for multiple-components LR, including Fu39-2 (20 ~ 50 ng), two entry clones (120 ~ 150ng, respectively) and 1 μL LR Clonase II Enzyme mix (Invitrogen). Incubate the mixture at 25°C for overnight and transform the LR reaction product to *E. coli* strain DH5α for selecting the positive clones through PCR.

### Transient expression in Arabidopsis protoplasts

*Arabidopsis* (Col) mesophyll protoplasts isolation and transformation were performed according to Sheen’s protocol (http://genetics.mgh.harvard.edu/sheenweb/faq.html). Briefly, well-expanded leaves of 3- to 4-week-old *Arabidopsis* plants grown on soil were cut into small strips with a razor blade and incubated in 10 mL of enzyme solution (0.4% Macerozyme R-10, 1.5% Cellulase R-10, 400 mM mannitol, 10 mM CaCl_2_, and 20 mM MES pH5.6, 20 mM KCl) at 23°C for 3 ~ 4 hours. After incubation, the protoplast suspension was filtered through 100 μm mesh and protoplasts were collected by centrifugation at 100xg for 2 min at 4°C. Wash the pelleted protoplasts once in cold W5 solution (154 mM NaCl, 125 mM CaCl_2_, 5 mM KCl, 2 mM MES pH5.7) and resuspend protoplasts in the same solution. Keep the protoplasts on ice for 30 min. Spin down protoplasts and resuspend in MMg solution (400 mM mannitol, 15 mM MgCl_2_, and 4 mM MES pH5.6) at a density of 1-2 × 10^5^ protoplasts/mL before PEG transfection. Warm the cold W5 solution to 23°C. To transform DNA into protoplasts, 20 μL plasmid DNA (about 20 μg) was added to 100 μL of protoplast suspension, mixed gently, then added 120 μL of PEG solution (200 mM mannitol, 100 mM CaCl_2_, and 40% PEG4000). The mixture was mixed gently and incubated for 30 min at room temperature. After incubation, the mixture was diluted with 500 μL W5 solution and spun at 100× g for 3 min at 23°C. The recovered protoplasts were resuspended in 1 mL W5 solution and incubated at 23°C in the dark for 12 ~ 14 hours.

### Transformation of arabidopsis (flower dipping)

After LR reaction, the resulted BDV was introduced into *Agrobacterium tumefaciens* strain GV3101 with pSoup by electroporation. Prepare the *A. tumefaciens* strain GV3101 carrying the wanted BDV by inoculating a single colony into 5 mL liquid LB medium containing the appropriate antibiotics for binary vector selection. Incubate culture at 28°C for 1 day. Use this feeder culture to inoculate a 100 mL liquid LB with the appropriate antibiotics and grow the culture at 28°C for overnight until the cells grown to OD_600_ = 1.5 ~ 2.0. Collected *Agrobacterium* cells by centrifugation at 4,000xg for 10 min at room temperature, and gently resuspended the pellet in 100 mL freshly prepared Dipping Buffer (5% sucrose, 10 mM MgCl_2_, 0.02% Silwet L-77). The inflorescences of *Arabidopsis* plants were submerged in the *Agrobacterium* cell suspension for 20 seconds and then bagged with plastic for 24 hours. After this incubation, plastic bags were removed. The transformed *Arabidopsis* plants were grown under LD in the greenhouse until seeds could be collected.

### Infiltration of nicotiana benthamiana leaf

*A. tumefaciens* strain EHA105 harboring the Fu39-8-*Ub*::*LUC* vector was grown at 28°C in LB medium supplemented with appropriate antibiotics to stationary phase. Bacteria were pelleted by centrifugation at 4000× g for 10 min at room temperature and wash the pellet once in Infiltration Buffer (10 mM MES pH5.6, 10 mM MgCl_2_, and 150 μM acetosyringone). Recover the bacteria by centrifugation at 4000× g for 10 min at room temperature and resuspend the cells with Infiltration Buffer to a density at OD600 = 1.0 ~ 1.5. Incubate this cell suspension at room temperature for 3 hours and then infiltrated into the abaxial side of 2- to 4-week-old *N. benthamiana* leaves. Samples could be collected after 3 days.

### Protein extraction and immunoblot analysis

After incubation for 12 ~ 14 h, the transformed *Arabidopsis* protoplasts were collected by centrifugation at 100xg for 2 min at room temperature. For extraction the total protein, the pelleted cells were incubated in 2× SDS loading buffer (100 mM Tris-HCl pH6.8, 4% SDS, 200 mM DTT, 0.2% bromophenol blue and 20% glycerol) at 95°C for 5 min, and then spun the mixture at 12,000x g for 10 min at room temperature. The extracts were loaded on 12% SDS-PAGE for separation. After electrophoresis, proteins were transferred onto ECL nitrocellulose membrane (GE Healthcare, no. RPN303D) by wet electroblotting. For detection of GFP, a mouse monoclonal GFP antibody (Roche, no. 11814460001) and a goat anti-mouse antibody conjugated to peroxidase (Pierce, no. 31430) were used at 1: 3000 and 1: 5000 dilutions, respectively. Blots were developed using the ECL kit (Pierce, no. 34079) and chemiluminescence emitted from the filter was visualized by ChemiDoc-It imaging System (UVP, Cambridge, UK).

### GUS histochemical staining

For GUS staining, seedlings were incubated in staining solution [ 20% methanol, 0.5 mg/ml X-Gluc (5-bromo-4-chloro-3-indolyl-β-D-glucuronide), 50 mM sodium phosphate buffer pH 7.0, 0.5 mM potassium ferrocyanide, 0.5 mM potassium ferricyanide, 0.1% Triton X-100] for overnight at 37°C. After staining, samples were washed once with 50 mM sodium phosphate buffer (pH 7.0) and cleared in 70% Ethanol. The GUS histochemical staining was visualized under a light stereomicroscope (Olympus, SZ2-ILST).

### Confocal microscopy

Localization of fluorescent proteins in protoplasts was visualized using a Leica TCSSP5 confocal laser scanning microscope. Water immersion objective lens with appropriate laser and filter combinations as follows: the 458 nm laser line with 470 to 500 nm band-pass emission filter for CFP; 488 nm laser line with a 505 to 520 nm band-pass emission filter for GFP; 514 nm laser line with a 520 to 540 nm band-pass emission filter for YFP; 543 nm laser line with a 580 to 630 nm band-pass emission filter for mRFP; 650 to 750 nm emission wavelengths for chloroplast autofluorescence. Bright field images were recorded by a transmission detector. The images were processed with Leica LAS AF and MacBiophotonics ImageJ softwares (http://rsbweb.nih.gov/ij/plugins/mbf/index.html).

### Ethanol induction and luciferase imaging

For testing the ethanol-inducible luciferase gene expression, the infiltrated *N. benthamiana* leaves were cut into pieces and imbedded in 1/2 MS liquid medium supplemented with (or without) 2% (v/v) ethanol for indicated hours. After incubation, the luciferin and Triton X-100 were added into the medium to a final concentration at 100 μM and 0.1%, respectively, the bioluminescence signals were imaged by a Princeton Instruments Digital CCD camera.

## Abbreviations

BDV: Binary destination vector; EC: Entry clones; GEC: Gene entry clone; NES: Nuclear export signal; NLS: Nuclear localization signal; PEC: Promoter entry clone; REL: Restriction enzyme/ligase; SSR: Site-specific recombination; SUC2: *SUCROSE H*^+^*SYMPORTER2*.

## Competing interests

The authors declare that they have no competing interests.

## Authors’ contributions

X W performed main experiments, analyzed the data, and wrote Methods; C F, X Z and J Z participated in some work; Y-F F conceived and supervised BioVector strategies, analyzed the data, and wrote the main manuscript. All authors read and approved the final manuscript.

## Supplementary Material

Additional file 1 The sequences of vectors, tags/markers, enhancers, resistant genes, and recombination sites. Click here for file
