# Supplementary material for: BioVector, a flexible system for gene specific-expression in plants
Source: BMC Plant Biol. 2013 Dec 5;13:198. doi: 10.1186/1471-2229-13-198 (PMC4235170; doi:10.1186/1471-2229-13-198)
Supplement: Additional file 1 — The sequences of vectors, tags/markers, enhancers, resistant genes, and recombination sites. [file 1471-2229-13-198-S1.doc]

**The sequences of vectors, tags/markers, enhancer, resistant gene, and recombination sites**

1. **Sequences of vectors**
   1. GEC (3013bp)：

tcatgacaaataatgattttattttgactgatagtgacctgttcgttgcaacacattgatgagcaatgcttttttataatgccaactttgtacaaaaaagcaggctttagatcttctagagatatcactagtctcgagccatggctgcagaagcttcatatggactttaggtccgactcactataggggaattgtgagcggataacaattcccctctaagaaataattttgtttaactttaagaaggagatataatgcagtttaaggtttacacctataaaagagagagccgttatcgtctgtttgtggatgtacagagtgatattattgacacgcccgggcgacggatggtgatccccctggccagtgcacgtctgctgtcagataaagtcccccgtgaactttacccggtggtgcatatcggggatgaaagctggcgcatgatgaccaccgatatggccagtgtgccggtctccgttatcggggaagaagtggctgatctcagccaccgcgaaaatgacatcaaaaacgccattaacctgatgttctggggaatataaaaaaaacccctcaagacccgtttagaggccccaaggggttatgctagacctaaagtcgcatgcggatccggtaccgtcgacattaatatgcattgcgcagaattcgagctctaagtagctgaacccagctttcttgtacaaagttggcattataagaaagcattgcttatcaatttgttgcaacgaacaggtcactatcagtcaaaataaaatcattatttgcgggcccttctatagtgtcacctaaatcgtatgcactggccgtcgttttacaacgtcgtgactgggaaaaccctggcgcttaagtaactaactaacaggaagagtttgtagaaacgcaaaaaggccatccgtcaggatggccttctgcttagtttgatgcctggcagtttatggcgggcgtcctgcccgccaccctccgggccgttgcttcacaacgttcaaatccgctcccggcggatttgtcctactcaggagagcgttcaccgacaaacaacagataaaacgaaaggcccagtcttccgactgagcctttcgttttatttgatgcctggcagttccctactctcgcttagtagttagacgtccccgagatccatgctagcggtaatacggttatccacagaatcaggggataacgcaggaaagaacatgtgagcaaaaggccagcaaaaggccaggaaccgtaaaaaggccgcgttgctggcgtttttccataggctccgcccccctgacgagcatcacaaaaatcgacgctcaagtcagaggtggcgaaacccgacaggactataaagataccaggcgtttccccctggaagctccctcgtgcgctctcctgttccgaccctgccgcttaccggatacctgtccgcctttctcccttcgggaagcgtggcgctttctcatagctcacgctgtaggtatctcagttcggtgtaggtcgttcgctccaagctgggctgtgtgcacgaaccccccgttcagcccgaccgctgcgccttatccggtaactatcgtcttgagtccaacccggtaagacacgacttatcgccactggcagcagccactggtaacaggattagcagagcgaggtatgtaggcggtgctacagagttcttgaagtggtggcctaactacggctacactagaagaacagtatttggtatctgcgctctgctgaagccagttaccttcggaaaaagagttggtagctcttgatccggcaaacaaaccaccgctggtagcggtggtttttttgtttgcaagcagcagattacgcgcagaaaaaaaggatctcaagaagatcctttgatcttttctaccgggtctgacgctcagtggaacggggcgggcggcgttgtgacaatttaccgaacaactccgcgggacttgacct gatagtttggctgtgagcaattatgtgcttagtgcatctaatcgcttgagttaacgccggcgaagcggcgtcggcttgaacgaatttctagctagaggatcgcaccaataactgccttaaaaaaattacgccccgccctgccactcatcgcagtactgttgtaattcattaagcattctgccgacatggaagccatcacaaacggcatgatgaacctgaatcgccagcggcatcagcaccttgtcgccttgcgtataatatttgcccattgtgaaaacgggggcgaagaagttgtccatattggccacgtttaaatcaaaactggtgaaactcacccagggattggctgagacgaaaaacatattctcaataaaccctttagggaaataggccaggttttcaccgtaacacgccacatcttgcgaatatatgtgtagaaactgccggaaatcgtcgtggtattcactccagagcgatgaaaacgtttcagtttgctcatggaaaacggtgtaacaagggtgaacactatcccatatcaccagctcaccgtctttcattgccatacggaactccggatgagcattcatcaggcgggcaagaatgtgaataaaggccggataaaacttgtgcttatttttctttacggtctttaaaaaggccgtaatatccagctgaacggtctggttataggtacattgagcaactgactgaaatgcctcaaaatgttctttacgatgccattgggatatatcaacggtggtatatccagtgatttttttctccatgatgtttaactttgttttagggcgactgccctgctgcgtaacatcgttgctgctccataacatcaaacatcgacccacggcgtaacgcgcttgctgcttggatgcccgaggcatagactgtaccccaaaaaaacatgtcataacaagaagccatgaaaaccgccactgcgccgttaccaccgctgcgttcggtcaaggttctggaccagttgcgtgacggcagttacgctacttgcattacagcttacgaaccgaacgaggcttatgtccac

- 1. PEC (3221bp)：

tcatgaaaataatgattttattttgactgatagtgacctgttcgttgcaacaaattgataagcaatgcttttttataatgccaactttgtataataaagttgaggcctcatatggtcgacatgcatgaattctctagagcatgcctcgaggagctcccatggcgtacggactttaggtcctggcagtccttgccattgccgggatcggggcagtaacgggatgggcgatcagcccgacaagctacccctatttgtttatttttctaaatacattcaaatatgtatccgctcaatgagacaataaccctgataaatgcttcaataatattgaaaaaggaagagtatgcagtttaaggtttacacctataaaagagagagccgttatcgtctgtttgtggatgtacagagtgatattattgacacgccaggacgacggatggtgatccccctggccagtgcacgtctgctgtcagataaagtcccccgtgaactttacccggtggtgcatatcggggatgaaagctggcgcatgatgaccaccgatatggccagtgtgccggtctccgttatcggggaagaagtggctgatctcagccaccgcgaaaatgacatcaaaaacgccattaacctgatgttctggggaatataactgtcagaccaagtttactcatatatactttagattgatttaaaacttcatttttaatttaaaaggatcataaaacgaaaggctcagtcggaagactgggcctttcgttttatagaaggccatcctgacggatggccttttgacctaaagtctacgtaactagtggtaccgatatcctgcagattaatcaattgcccgggggatccaagcttgcggccgcagatctacgcgttgcgcaatttaaatcaacttttctatacaaagttggcattataaaaaagcattgcttatcaatttgttgcaacgaacaggtcactatcagtcaaaataaaatcattatttgggcccttctatagtgtcacctaaatcgtatgcactggccgtcgttttacaacgtcgtgactgggaaaaccctggcgcttaagtaactaactaacaggaagagtttgtagaaacgcaaaaaggccatccgtcaggatggccttctgcttagtttgatgcctggcagtttatggcgggcgtcctgcccgccaccctccgggccgttgcttcacaacgttcaaatccgctcccggcggatttgtcctactcaggagagcgttcaccgacaaacaacagataaaacgaaaggcccagtcttccgactgagcctttcgttttatttgatgcctggcagttccctactctcgcttagtagttagacgtccccgagatccatgctagcggtaatacggttatccacagaatcaggggataacgcaggaaagaacatgtgagcaaaaggccagcaaaaggccaggaaccgtaaaaaggccgcgttgctggcgtttttccataggctccgcccccctgacgagcatcacaaaaatcgacgctcaagtcagaggtggcgaaacccgacaggactataaagataccaggcgtttccccctggaagctccctcgtgcgctctcctgttccgaccctgccgcttaccggatacctgtccgcctttctcccttcgggaagcgtggcgctttctcatagctcacgctgtaggtatctcagttcggtgtaggtcgttcgctccaagctgggctgtgtgcacgaaccccccgttcagcccgaccgctgcgccttatccggtaactatcgtcttgagtccaacccggtaagacacgacttatcgccactggcagcagccactggtaacaggattagcagagcgaggtatgtaggcggtgctacagagttcttgaagtggtggcctaactacggctacactagaagaacagtatttggtatctgcgctctgctgaagccagttaccttcggaaaaagagttggtagctcttgatccggcaaacaaaccaccgctggtagcggtggtttttttgtttgcaagcagcagattacgcgcagaaaaaaaggatctcaagaagatcctttgatcttttctaccgggtctgacgctcagtggaacggggcgggcggcgttgtgacaatttaccgaacaactccgcgggacttgacctgatagtttggctgtgagcaattatgtgcttagtgcatctaatcgcttgagttaacgccggcgaagcggcgtcggcttgaacgaatttctagctagaggatcgcaccaataactgccttaaaaaaattacgccccgccctgccactcatcgcagtactgttgtaattcattaagcattctgccgacatggaagccatcacaaacggcatgatgaacctgaatcgccagcggcatcagcaccttgtcgccttgcgtataatatttgcccattgtgaaaacgggggcgaagaagttgtccatattggccacgtttaaatcaaaactggtgaaactcacccagggattggctgagacgaaaaacatattctcaataaaccctttagggaaataggccaggttttcaccgtaacacgccacatcttgcgaatatatgtgtagaaactgccggaaatcgtcgtggtattcactccagagcgatgaaaacgtttcagtttgctcatggaaaacggtgtaacaagggtgaacactatcccatatcaccagctcaccgtctttcattgccatacggaactccggatgagcattcatcaggcgggcaagaatgtgaataaaggccggataaaacttgtgcttatttttctttacggtctttaaaaaggccgtaatatccagctgaacggtctggttataggtacattgagcaactgactgaaatgcctcaaaatgttctttacgatgccattgggatatatcaacggtggtatatccagtgatttttttctccatgatgtttaactttgttttagggcgactgccctgctgcgtaacatcgttgctgctccataacatcaaacatcgacccacggcgtaacgcgcttgctgcttggatgcccgaggcatagactgtaccccaaaaaaacatgtcataacaagaagccatgaaaaccgccactgcgccgttaccaccgctgcgttcggtcaaggttctggaccagttgcgtgacggcagttacgctacttgcattacagcttacgaaccgaacgaggcttatgtccac

- 1. BDV1 (4972bp)：

agatcttggcaggatatattgtggtgtaaacgttcctgcggcggtcgagatggatcttggcaggatatattgtggtgtaaacacgcgtatcagcttgcgatgccggtcgatctagtaacatagatgacaccgcgcgcgataatttatcctagtttgcgcgctatattttgttttctatcgcgtattaaatgtataattgcgggactctaatcataaaaacccatctcataaataacgtcatgcattacatgttaattattacatgcttaacgtaattcaacagaaattatatgataatcatcgcaagaccggcaacaggattcaatcttaagaaactttattgccaaatgtttgaacgatctgcttgactctagtatttaaatgggcccgcggccgctcagatttcggtgacgggcaggaccggacggggcggcaccggcaggctgaagtccagctgccagaaacccacgtcatgccagttcccgtgcttgaagccggccgcccgcagcattccgcggggggcatatccgagcgcctcgtgcattcgcacgctcgggtcgttgggcagcccgatgacagcgaccacgctcttgaagccctgtgcctccagggacttcagcaggtgggtgtagagcgtggagcccagtcccgtccgctggtggcggggggagacgtacacggttgactcggccgtccagtcgtaggcgttgcgtgccttccagggtcccgcgtaggcgatgccggcgacctcgccgtccacctcggcgacgagccagggatagcgctcccgcagacggacgaggtcgtccgtccactcctgcggttcctgcggctcggtacggaagttgaccgtgcttgtctcgatgtagtggttgacgatggtgcagaccgccggcatgtccgcctcggtggcacggcggatgtcggccgggcgtcgttctgggctcatcccggggacgtcgtcgacttgagagtgaatatgagactctaattggataccgaggggaatttatggaacgtcagtggagcatttttgacaagaaatatttgcgtagctgatagtgaccttaggcgacttttgaacgcgcaataatggtttctgacgtatgtgcttagctcattaaactccagaaacccgcggctgagtggctccttcaacgttgcggttctgtcagttccaaacgtaaaacggcttgtcccgcgtcatcggcgggggtcataacgtgactcccttaattctccgctcatgatcagattgtcgtttcccgccttcggtttgggcgcgccgttaacaccggaggatccacaagtttgtacaaaaaagctgaacgagaaacgtaaaatgatataaatatcaatatattaaattagattttgcataaaaaacagactacataatactgtaaaacacaacatatccagtcactatgtaatacgactcactatagggagaccacaacggtttccctctagaaataattttgtttaactttaagaaggagatatacatatgatgcagtttaaggtttacacctataaaagagagagccgttatcgtctgtttgtggatgtacagagtgatattattgacacgcccgggcgacggatggtgatccccctggccagtgcacgtctgctgtcagataaagtcccccgtgaactttacccggtggtgcatatcggggatgaaagctggcgcatgatgaccaccgatatggccagtgtgccggtctccgttatcggggaagaagtggctgatctcagccaccgcgaaaatgacatcaaaaacgccattaacctgatgttctggggaatataactgctaacaaagcccgaaaggaagctgagttggctgctgccaccgctgagcaataactagcataaccccttggggcctctaaacgggtcttgaggggttttttgctgaaaggaggaactatatccggatcatagtgactggatatgttgtgttttacagtattatgtagtctgttttttatgcaaaatctaatttaatatattgatatttatatcattttacgtttctcgttcagctttcttgtacaaagtggtctgcagtaattgattagtcctagagtccgcaaaaatcaccagtctctctctacaaatctatctctctctatttttctccagaataatgtgtgagtagttcccagataagggaattagggttcttatagggtttcgctcatgtgttgagcatataagaaacccttagtatgtatttgtatttgtaaaatacttctatcaataaaatttctaattcctaaaaccaaaatccagtgaccttaattaatgacaggatatattggcgggtaaactaagtcgctgtatgtgtttgtttgcgatcgcatgtgagcaaaaggccagcaaaaggccaggaaccgtaaaaaggccgcgttgctggcgtttttccataggctccgcccccctgacgagcatcacaaaaatcgacgctcaagtcagaggtggcgaaacccgacaggactataaagataccaggcgtttccccctggaagctccctcgtgcgctctcctgttccgaccctgccgcttaccggatacctgtccgcctttctcccttcgggaagcgtggcgctttctcatagctcacgctgtaggtatctcagttcggtgtaggtcgttcgctccaagctgggctgtgtgcacgaaccccccgttcagcccgaccgctgcgccttatccggtaactatcgtcttgagtccaacccggtaagacacgacttatcgccactggcagcagccactggtaacaggattagcagagcgaggtatgtaggcggtgctacagagttcttgaagtggtggcctaactacggctacactagaagaacagtatttggtatctgcgctctgctgaagccagttaccttcggaagaagagttggtagctcttgatccggcaaacaaaccaccgctggtagcggtggtttttttgtttgcaagcagcagattacgcgcagaaaaaaaggatctcaagaagatcctttgatcttttctacggggtctgacgctcagtggaacgaaaactcacgttaagggattttggtcatgagattatcaaaaaggatcttcacctagatccttttaaattaaaaatgaagttttaaatcaatctaaagtatatatgtgtaacattggtctagtgaatcgatttaccaatgcttaatcagtgaggcacctatctcagcgatctgtctatttcgttcatccatagttgcctgactccccgtcgtgtagataactacgatacgggagggcttaccatctggccccagtgctgcaatgataccgcgagacccacgctcaccggctccagatttatcagcaataaaccagccagccggaagggccgagcgcagaagtggtcctgcaactttatccgcctccatccagtctattaattgttgccgggaagctagagtaagtagttcgccagttaatagtttgcgcaacgttgttgccattgcagcaggcatcgtggtgtcacgctcgtcgtttggtatggcttcattcagctccggttcccaacgatcaaggcgagttacatgatcccccatgttgtgcaaaaaagcggttagctccttcggtcctcctatcgttgtcagaagtaagttggccgcagtgttatcactcatggttatggcagcactgcataattctcttactgtcatgccatccgtaagatgcttttctgtgactggtgaatactcaaccaagtcattctgagaatagtgtatgcggcgaccgagttgctcttgcccggcgtcaacacgggataataccgcgccacatagcagaactttaaaagtgctcatcattggaaaacgttcttcggggcgaaaactctcaaggatcttaccgctgttgagatccagttcgatgtaacccactcgtgcacccaactgatcttcagcatcttttactttcaccagcgtttctgggtgagcaaaaacaggaaggcaaaatgccgcaaaaaagggaataagggcgacacggaaatgttgaatactcatgctagcgttggaatttaatcgcggccttgagcaagacgtttcccgttgaatatggctcataacaccccttgtattactgtttatgtaagcagacagttttattgttcatgatgatatatttttatcttgtgcaatgtaacatcagagattttgagacacaacgtggctttgttgaataaatcgaacttttgctgagttgaaggatcagatcacgcatcttcccgacaacgcagaccgttccgtggcaaagcaaaagttcaaaatcaccaactggtccacctacaacaaagctctcatcaaccgtggctccctcactttctggctggatgatggggcgattcaggcgatccccatccaacagcccgccgtcgagcgggcttttttatccccggaagcctgtggatagagggtagttatccacgtgaaaccgctaatgccccgcaaagccttgattcacggggctttccggcccgctccaaaaactatccacgtgaaatcgctaatcagggtacgtgaaatcgctaatcggagtacgtgaaatcgctaataaggtcacgtgaaatcgctaatcaaaaaggcacgtgagaacgctaatagccctttcagatcaacagcttgcaaacacccctcgctccggcaagtagttacagcaagtagtatgttcaattagcttttcaattatgaatatatatatcaattattggtcgcccttggcttgtggacaatgcgctacgcgcaccggctccgcccgtggacaaccgcaagcggttgcccaccgtcgagcgccagcgcctttgcccacaacccggcggccggccgcaacagatcgttttataaatttttttttttgaaaaagaaaaagcccgaaaggcggcaacctctcgggcttctggatttccgatccccggaattag

- 1. BDV2 (5812bp)：

agatcttggcaggatatattgtggtgtaaacgttcctgcggcggtcgagatggatcttggcaggatatattgtggtgtaaacacgcgtatcagcttgcgatgccggtcgatctagtaacatagatgacaccgcgcgcgataatttatcctagtttgcgcgctatattttgttttctatcgcgtattaaatgtataattgcgggactctaatcataaaaacccatctcataaataacgtcatgcattacatgttaattattacatgcttaacgtaattcaacagaaattatatgataatcatcgcaagaccggcaacaggattcaatcttaagaaactttattgccaaatgtttgaacgatctgcttgactctagtatttaaatgggcccgcggccgctcagatttcggtgacgggcaggaccggacggggcggcaccggcaggctgaagtccagctgccagaaacccacgtcatgccagttcccgtgcttgaagccggccgcccgcagcattccgcggggggcatatccgagcgcctcgtgcattcgcacgctcgggtcgttgggcagcccgatgacagcgaccacgctcttgaagccctgtgcctccagggacttcagcaggtgggtgtagagcgtggagcccagtcccgtccgctggtggcggggggagacgtacacggttgactcggccgtccagtcgtaggcgttgcgtgccttccagggtcccgcgtaggcgatgccggcgacctcgccgtccacctcggcgacgagccagggatagcgctcccgcagacggacgaggtcgtccgtccactcctgcggttcctgcggctcggtacggaagttgaccgtgcttgtctcgatgtagtggttgacgatggtgcagaccgccggcatgtccgcctcggtggcacggcggatgtcggccgggcgtcgttctgggctcatcccggggacgtcgtcgacttgagagtgaatatgagactctaattggataccgaggggaatttatggaacgtcagtggagcatttttgacaagaaatatttgcgtagctgatagtgaccttaggcgacttttgaacgcgcaataatggtttctgacgtatgtgcttagctcattaaactccagaaacccgcggctgagtggctccttcaacgttgcggttctgtcagttccaaacgtaaaacggcttgtcccgcgtcatcggcgggggtcataacgtgactcccttaattctccgctcatgatcagattgtcgtttcccgccttcggtttgggcgcgccgttaacaccggtaggcctaactatgtataataaagttgaacgagaaacgtaaaatgatataaatatcaatatattaaattagattttgcataaaaaacagactacataatactgtaaaacacaacatatccagtcactatggcctcagcatttaaataatacgactcactatagggagaccacaacggtttccctctagaaataattttgtttaactttaagaaggagatatacatatgatgcagtttaaggtttacacctataaaagagagagccgttatcgtctgtttgtggatgtacagagtgatattattgacacgcccgggcgacggatggtgatccccctggccagtgcacgtctgctgtcagataaagtcccccgtgaactttacccggtggtgcatatcggggatgaaagctggcgcatgatgaccaccgatatggccagtgtgccggtctccgttatcggggaagaagtggctgatctcagccaccgcgaaaatgacatcaaaaacgccattaacctgatgttctggggaatataactgctaacaaagcccgaaaggaagctgagttggctgctgccaccgctgagcaataactagcataaccccttggggcctctaaacgggtcttgaggggttttttgctgaaaggaggaactatatccggatccatagtgactggatatgttgtgttttacagtattatgtagtctgttttttatgcaaaatctaatttaatatattgatatttatatcattttacgtttctcgttcaacttttctatacaaagttgaaattaaaactcgttataaagacaacaaaggcgcccaattgttcgaatccggaggatccacaagtttgtacaaaaaagctgaacgagaaacgtaaaatgatataaatatcaatatattaaattagattttgcataaaaaacagactacataatactgtaaaacacaacatatccagtcactatgtaatacgactcactatagggagaccacaacggtttccctctagaaataattttgtttaactttaagaaggagatatacatatgatgcagtttaaggtttacacctataaaagagagagccgttatcgtctgtttgtggatgtacagagtgatattattgacacgcccgggcgacggatggtgatccccctggccagtgcacgtctgctgtcagataaagtcccccgtgaactttacccggtggtgcatatcggggatgaaagctggcgcatgatgaccaccgatatggccagtgtgccggtctccgttatcggggaagaagtggctgatctcagccaccgcgaaaatgacatcaaaaacgccattaacctgatgttctggggaatataactgctaacaaagcccgaaaggaagctgagttggctgctgccaccgctgagcaataactagcataaccccttggggcctctaaacgggtcttgaggggttttttgctgaaaggaggaactatatccggatcatagtgactggatatgttgtgttttacagtattatgtagtctgttttttatgcaaaatctaatttaatatattgatatttatatcattttacgtttctcgttcagctttcttgtacaaagtggtctgcagtaattgattagtcctagagtccgcaaaaatcaccagtctctctctacaaatctatctctctctatttttctccagaataatgtgtgagtagttcccagataagggaattagggttcttatagggtttcgctcatgtgttgagcatataagaaacccttagtatgtatttgtatttgtaaaatacttctatcaataaaatttctaattcctaaaaccaaaatccagtgaccttaattaatgacaggatatattggcgggtaaactaagtcgctgtatgtgtttgtttgcgatcgcatgtgagcaaaaggccagcaaaaggccaggaaccgtaaaaaggccgcgttgctggcgtttttccataggctccgcccccctgacgagcatcacaaaaatcgacgctcaagtcagaggtggcgaaacccgacaggactataaagataccaggcgtttccccctggaagctccctcgtgcgctctcctgttccgaccctgccgcttaccggatacctgtccgcctttctcccttcgggaagcgtggcgctttctcatagctcacgctgtaggtatctcagttcggtgtaggtcgttcgctccaagctgggctgtgtgcacgaaccccccgttcagcccgaccgctgcgccttatccggtaactatcgtcttgagtccaacccggtaagacacgacttatcgccactggcagcagccactggtaacaggattagcagagcgaggtatgtaggcggtgctacagagttcttgaagtggtggcctaactacggctacactagaagaacagtatttggtatctgcgctctgctgaagccagttaccttcggaagaagagttggtagctcttgatccggcaaacaaaccaccgctggtagcggtggtttttttgtttgcaagcagcagattacgcgcagaaaaaaaggatctcaagaagatcctttgatcttttctacggggtctgacgctcagtggaacgaaaactcacgttaagggattttggtcatgagattatcaaaaaggatcttcacctagatccttttaaattaaaaatgaagttttaaatcaatctaaagtatatatgtgtaacattggtctagtgaatcgatttaccaatgcttaatcagtgaggcacctatctcagcgatctgtctatttcgttcatccatagttgcctgactccccgtcgtgtagataactacgatacgggagggcttaccatctggccccagtgctgcaatgataccgcgagacccacgctcaccggctccagatttatcagcaataaaccagccagccggaagggccgagcgcagaagtggtcctgcaactttatccgcctccatccagtctattaattgttgccgggaagctagagtaagtagttcgccagttaatagtttgcgcaacgttgttgccattgcagcaggcatcgtggtgtcacgctcgtcgtttggtatggcttcattcagctccggttcccaacgatcaaggcgagttacatgatcccccatgttgtgcaaaaaagcggttagctccttcggtcctcctatcgttgtcagaagtaagttggccgcagtgttatcactcatggttatggcagcactgcataattctcttactgtcatgccatccgtaagatgcttttctgtgactggtgaatactcaaccaagtcattctgagaatagtgtatgcggcgaccgagttgctcttgcccggcgtcaacacgggataataccgcgccacatagcagaactttaaaagtgctcatcattggaaaacgttcttcggggcgaaaactctcaaggatcttaccgctgttgagatccagttcgatgtaacccactcgtgcacccaactgatcttcagcatcttttactttcaccagcgtttctgggtgagcaaaaacaggaaggcaaaatgccgcaaaaaagggaataagggcgacacggaaatgttgaatactcatgctagcgttggaatttaatcgcggccttgagcaagacgtttcccgttgaatatggctcataacaccccttgtattactgtttatgtaagcagacagttttattgttcatgatgatatatttttatcttgtgcaatgtaacatcagagattttgagacacaacgtggctttgttgaataaatcgaacttttgctgagttgaaggatcagatcacgcatcttcccgacaacgcagaccgttccgtggcaaagcaaaagttcaaaatcaccaactggtccacctacaacaaagctctcatcaaccgtggctccctcactttctggctggatgatggggcgattcaggcgatccccatccaacagcccgccgtcgagcgggcttttttatccccggaagcctgtggatagagggtagttatccacgtgaaaccgctaatgccccgcaaagccttgattcacggggctttccggcccgctccaaaaactatccacgtgaaatcgctaatcagggtacgtgaaatcgctaatcggagtacgtgaaatcgctaataaggtcacgtgaaatcgctaatcaaaaaggcacgtgagaacgctaatagccctttcagatcaacagcttgcaaacacccctcgctccggcaagtagttacagcaagtagtatgttcaattagcttttcaattatgaatatatatatcaattattggtcgcccttggcttgtggacaatgcgctacgcgcaccggctccgcccgtggacaaccgcaagcggttgcccaccgtcgagcgccagcgcctttgcccacaacccggcggccggccgcaacagatcgttttataaatttttttttttgaaaaagaaaaagcccgaaaggcggcaacctctcgggcttctggatttccgatccccggaattag

- 1. BDV3 (6819bp)：

agatcttggcaggatatattgtggtgtaaacgttcctgcggcggtcgagatggatcttggcaggatatattgtggtgtaaacacgcgtatcagcttgcgatgccggtcgatctagtaacatagatgacaccgcgcgcgataatttatcctagtttgcgcgctatattttgttttctatcgcgtattaaatgtataattgcgggactctaatcataaaaacccatctcataaataacgtcatgcattacatgttaattattacatgcttaacgtaattcaacagaaattatatgataatcatcgcaagaccggcaacaggattcaatcttaagaaactttattgccaaatgtttgaacgatctgcttgactctagtatttaaatgggcccgcggccgctcagatttcggtgacgggcaggaccggacggggcggcaccggcaggctgaagtccagctgccagaaacccacgtcatgccagttcccgtgcttgaagccggccgcccgcagcattccgcggggggcatatccgagcgcctcgtgcattcgcacgctcgggtcgttgggcagcccgatgacagcgaccacgctcttgaagccctgtgcctccagggacttcagcaggtgggtgtagagcgtggagcccagtcccgtccgctggtggcggggggagacgtacacggttgactcggccgtccagtcgtaggcgttgcgtgccttccagggtcccgcgtaggcgatgccggcgacctcgccgtccacctcggcgacgagccagggatagcgctcccgcagacggacgaggtcgtccgtccactcctgcggttcctgcggctcggtacggaagttgaccgtgcttgtctcgatgtagtggttgacgatggtgcagaccgccggcatgtccgcctcggtggcacggcggatgtcggccgggcgtcgttctgggctcatcccggggacgtcgtcgacttgagagtgaatatgagactctaattggataccgaggggaatttatggaacgtcagtggagcatttttgacaagaaatatttgcgtagctgatagtgaccttaggcgacttttgaacgcgcaataatggtttctgacgtatgtgcttagctcattaaactccagaaacccgcggctgagtggctccttcaacgttgcggttctgtcagttccaaacgtaaaacggcttgtcccgcgtcatcggcgggggtcataacgtgactcccttaattctccgctcatgatcagattgtcgtttcccgccttcggtttgggcgcgccgttaacaccggtaggcctaactatgtataataaagttgaacgagaaacgtaaaatgatataaatatcaatatattaaattagattttgcataaaaaacagactacataatactgtaaaacacaacatatccagtcactatggcctcagcatttaaataatacgactcactatagggagaccacaacggtttccctctagaaataattttgtttaactttaagaaggagatatacatatgatgcagtttaaggtttacacctataaaagagagagccgttatcgtctgtttgtggatgtacagagtgatattattgacacgcccgggcgacggatggtgatccccctggccagtgcacgtctgctgtcagataaagtcccccgtgaactttacccggtggtgcatatcggggatgaaagctggcgcatgatgaccaccgatatggccagtgtgccggtctccgttatcggggaagaagtggctgatctcagccaccgcgaaaatgacatcaaaaacgccattaacctgatgttctggggaatataactgctaacaaagcccgaaaggaagctgagttggctgctgccaccgctgagcaataactagcataaccccttggggcctctaaacgggtcttgaggggttttttgctgaaaggaggaactatatccggatccatagtgactggatatgttgtgttttacagtattatgtagtctgttttttatgcaaaatctaatttaatatattgatatttatatcattttacgtttctcgttcaacttttctatacaaagttgaaattaaaactcgttataaagacaacaaaggcgcccaattgttcgaatccggaggatccacaagtttgtacaaaaaagctgaacgagaaacgtaaaatgatataaatatcaatatattaaattagattttgcataaaaaacagactacataatactgtaaaacacaacatatccagtcactatgtaatacgactcactatagggagaccacaacggtttccctctagaaataattttgtttaactttaagaaggagatatacatatgatgcagtttaaggtttacacctataaaagagagagccgttatcgtctgtttgtggatgtacagagtgatattattgacacgcccgggcgacggatggtgatccccctggccagtgcacgtctgctgtcagataaagtcccccgtgaactttacccggtggtgcatatcggggatgaaagctggcgcatgatgaccaccgatatggccagtgtgccggtctccgttatcggggaagaagtggctgatctcagccaccgcgaaaatgacatcaaaaacgccattaacctgatgttctggggaatataactgctaacaaagcccgaaaggaagctgagttggctgctgccaccgctgagcaataactagcataaccccttggggcctctaaacgggtcttgaggggttttttgctgaaaggaggaactatatccggatcatagtgactggatatgttgtgttttacagtattatgtagtctgttttttatgcaaaatctaatttaatatattgatatttatatcattttacgtttctcgttcagctttcttgtacaaagtggtctgcagcaattgctcctcctctgctaacgtaagcctctctgttttttttctctgtttcttttgaaatgaatccaattagtgatgataatctgtgtttgatgtatcattgatttaacatcttgacaatgaatcgtgatcggaagtgataaagttatgggtcaacggtttcaaagagagagaaagacttttagagtcaactctcgactctttcttaattatgttattgctatttgtctcttttcttgaagtctgaacaattcttgggattgttttgcaggttctagcttctccaaccacagcaattgaccactttgtacaagaaagctgaacgagaaacgtaaaatgatataaatatcaatatattaaattagattttgcataaaaaacagactacataatactgtaaaacacaacatatccagtcactatgatccggatatagttcctcctttcagcaaaaaacccctcaagacccgtttagaggccccaaggggttatgctagttattgctcagcggtggcagcagccaactcagcttcctttcgggctttgttagcagttatattccccagaacatcaggttaatggcgtttttgatgtcattttcgcggtggctgagatcagccacttcttccccgataacggagaccggcacactggccatatcggtggtcatcatgcgccagctttcatccccgatatgcaccaccgggtaaagttcacgggggactttatctgacagcagacgtgcactggccagggggatcaccatccgtcgcccgggcgtgtcaataatatcactctgtacatccacaaacagacgataacggctctctcttttataggtgtaaaccttaaactgcatcatatgtatatctccttcttaaagttaaacaaaattatttctagagggaaaccgttgtggtctccctatagtgagtcgtattacatagtgactggatatgttgtgttttacagtattatgtagtctgttttttatgcaaaatctaatttaatatattgatatttatatcattttacgtttctcgttcagcttttttgtacaaacttgtctgcagtaattgattagtcctagagtccgcaaaaatcaccagtctctctctacaaatctatctctctctatttttctccagaataatgtgtgagtagttcccagataagggaattagggttcttatagggtttcgctcatgtgttgagcatataagaaacccttagtatgtatttgtatttgtaaaatacttctatcaataaaatttctaattcctaaaaccaaaatccagtgaccttaattaatgacaggatatattggcgggtaaactaagtcgctgtatgtgtttgtttgcgatcgcatgtgagcaaaaggccagcaaaaggccaggaaccgtaaaaaggccgcgttgctggcgtttttccataggctccgcccccctgacgagcatcacaaaaatcgacgctcaagtcagaggtggcgaaacccgacaggactataaagataccaggcgtttccccctggaagctccctcgtgcgctctcctgttccgaccctgccgcttaccggatacctgtccgcctttctcccttcgggaagcgtggcgctttctcatagctcacgctgtaggtatctcagttcggtgtaggtcgttcgctccaagctgggctgtgtgcacgaaccccccgttcagcccgaccgctgcgccttatccggtaactatcgtcttgagtccaacccggtaagacacgacttatcgccactggcagcagccactggtaacaggattagcagagcgaggtatgtaggcggtgctacagagttcttgaagtggtggcctaactacggctacactagaagaacagtatttggtatctgcgctctgctgaagccagttaccttcggaagaagagttggtagctcttgatccggcaaacaaaccaccgctggtagcggtggtttttttgtttgcaagcagcagattacgcgcagaaaaaaaggatctcaagaagatcctttgatcttttctacggggtctgacgctcagtggaacgaaaactcacgttaagggattttggtcatgagattatcaaaaaggatcttcacctagatccttttaaattaaaaatgaagttttaaatcaatctaaagtatatatgtgtaacattggtctagtgaatcgattcagaagaactcgtcaagaaggcgatagaaggcgatgcgctgcgaatcgggagcggcgataccgtaaagcacgaggaagcggtcagcccattcgccgccaagctcttcagcaatatcacgggtagccaacgctatgtcctgatagcggtccgccacacccagccggccacagtcgatgaatccagaaaagcggccattttccaccatgatattcggcaagcaggcatcgccatgggtcacgacgagatcatcgccgtcgggcatgcgcgccttgagcctggcgaacagttcggctggcgcgagcccctgatgctcttcgtccagatcatcctgatcgacaagaccggcttccatccgagtacgtgctcgctcgatgcgatgtttcgcttggtggtcgaatgggcaggtagccggatcaagcgtatgcagccgccgcattgcatcagccatgatggatactttctcggcaggagcaaggtgagatgacaggagatcctgccccggcacttcgcccaatagcagccagtcccttcccgcttcagtgacaacgtcgagcacagctgcgcaaggaacgcccgtcgtggccagccacgatagccgcgctgcctcgtcctgcagttcattcagggcaccggacaggtcggtcttgacaaaaagaaccgggcgcccctgcgctgacagccggaacacggcggcatcagagcagccgattgtctgttgtgcccagtcatagccgaatagcctctccacccaagcggccggagaacctgcgtgcaatccatcttgttcaatcatgctagcgttggaatttaatcgcggccttgagcaagacgtttcccgttgaatatggctcataacaccccttgtattactgtttatgtaagcagacagttttattgttcatgatgatatatttttatcttgtgcaatgtaacatcagagattttgagacacaacgtggctttgttgaataaatcgaacttttgctgagttgaaggatcagatcacgcatcttcccgacaacgcagaccgttccgtggcaaagcaaaagttcaaaatcaccaactggtccacctacaacaaagctctcatcaaccgtggctccctcactttctggctggatgatggggcgattcaggcgatccccatccaacagcccgccgtcgagcgggcttttttatccccggaagcctgtggatagagggtagttatccacgtgaaaccgctaatgccccgcaaagccttgattcacggggctttccggcccgctccaaaaactatccacgtgaaatcgctaatcagggtacgtgaaatcgctaatcggagtacgtgaaatcgctaataaggtcacgtgaaatcgctaatcaaaaaggcacgtgagaacgctaatagccctttcagatcaacagcttgcaaacacccctcgctccggcaagtagttacagcaagtagtatgttcaattagcttttcaattatgaatatatatatcaattattggtcgcccttggcttgtggacaatgcgctacgcgcaccggctccgcccgtggacaaccgcaagcggttgcccaccgtcgagcgccagcgcctttgcccacaacccggcggccggccgcaacagatcgttttataaatttttttttttgaaaaagaaaaagcccgaaaggcggcaacctctcgggcttctggatttccgatccccggaattag

- 1. BDV4 (8912bp)：

agatcttggcaggatatattgtggtgtaaacgttcctgcggcggtcgagatggatcttggcaggatatattgtggtgtaaacacgcgtatcagcttgcgatgccggtcgatctagtaacatagatgacaccgcgcgcgataatttatcctagtttgcgcgctatattttgttttctatcgcgtattaaatgtataattgcgggactctaatcataaaaacccatctcataaataacgtcatgcattacatgttaattattacatgcttaacgtaattcaacagaaattatatgataatcatcgcaagaccggcaacaggattcaatcttaagaaactttattgccaaatgtttgaacgatctgcttgactctagtatttaaatgggcccgcggccgctcagatttcggtgacgggcaggaccggacggggcggcaccggcaggctgaagtccagctgccagaaacccacgtcatgccagttcccgtgcttgaagccggccgcccgcagcattccgcggggggcatatccgagcgcctcgtgcattcgcacgctcgggtcgttgggcagcccgatgacagcgaccacgctcttgaagccctgtgcctccagggacttcagcaggtgggtgtagagcgtggagcccagtcccgtccgctggtggcggggggagacgtacacggttgactcggccgtccagtcgtaggcgttgcgtgccttccagggtcccgcgtaggcgatgccggcgacctcgccgtccacctcggcgacgagccagggatagcgctcccgcagacggacgaggtcgtccgtccactcctgcggttcctgcggctcggtacggaagttgaccgtgcttgtctcgatgtagtggttgacgatggtgcagaccgccggcatgtccgcctcggtggcacggcggatgtcggccgggcgtcgttctgggctcatcccggggacgtcgtcgacttgagagtgaatatgagactctaattggataccgaggggaatttatggaacgtcagtggagcatttttgacaagaaatatttgcgtagctgatagtgaccttaggcgacttttgaacgcgcaataatggtttctgacgtatgtgcttagctcattaaactccagaaacccgcggctgagtggctccttcaacgttgcggttctgtcagttccaaacgtaaaacggcttgtcccgcgtcatcggcgggggtcataacgtgactcccttaattctccgctcatgatcagattgtcgtttcccgccttcggtttgggcgcgccaactatgtataataaagttgaacgagaaacgtaaaatgatataaatatcaatatattaaattagattttgcataaaaaacagactacataatactgtaaaacacaacatatccagtcactatggcctcagcatttaaataatacgactcactatagggagaccacaacggtttccctctagaaataattttgtttaactttaagaaggagatatacatatgatgcagtttaaggtttacacctataaaagagagagccgttatcgtctgtttgtggatgtacagagtgatattattgacacgcccgggcgacggatggtgatccccctggccagtgcacgtctgctgtcagataaagtcccccgtgaactttacccggtggtgcatatcggggatgaaagctggcgcatgatgaccaccgatatggccagtgtgccggtctccgttatcggggaagaagtggctgatctcagccaccgcgaaaatgacatcaaaaacgccattaacctgatgttctggggaatataagtttaaacgcgctagcttaattaatacgtacggccggggcccgctcagcctgctaacaaagcccgaaaggaagctgagttggctgctgccaccgctgagcaataactagcataaccccttggggcctctaaacgggtcttgaggggttttttgctgaaaggaggaactatatccggatccatagtgactggatatgttgtgttttacagtattatgtagtctgttttttatgcaaaatctaatttaatatattgatatttatatcattttacgtttctcgttcaacttttctatacaaagttgaaattaaaactcgttataaagacaacaaactcgagatggcagatacgcgccgacgccagaatcatagctgcgatccctgtcgcaagggcaagcgacgctgtgatgccccggaaaatagaaacgaggccaatgaaaacggctgggtttcgtgttcaaattgcaagcgttggaacaaggattgtaccttcaactggctctcatcccaacgctccaaggcaaaaggggctgcacctagagcgagaacaaagaaagccaggaccgcaacaaccaccagtgaaccatcaacttcagctgcaacaatccctacaccggaaagtgacaatcacgatgcgcctccagtcataaactctcacgacgcgctcccgagctggactcaggggctactctcccaccccggcgaccttttcgatttcagccactctgctattcccgcaaatgcagaagatgcggccaacgtgcagtcagacgcaccttttccgtgggatctagccatccccggtgatttcagcatgggccaacagctggagaaacctctcagtccgctaagttttcaagcagtccttcttccgccccatagcccgaacacggatgacctcattcgcgagctggaagagcagactaccgatccggactcggttaccgatactaatagtgttcaacaggtcgctcaagatggatcgctatggtctgatcggcagtcgccgctactgcctgagaacagtctgtgcatggcctcagacagcacagcacggcgatatgcccgttccacaatgacgaagaatctgatgcgaatctaccacgatagtatggagaatgcactgtcctgctggctgacagagcacaactgtccatactccgaccagatcagctacctgccgcccaagcagcgggcggaatggggaccgaactggtcaaacaggatgtgcatccgggtgtgccggctagatcgcgtatctacctcattacgcgggcgtgcactgagtgcggaagaggacaaagccgcagcccgagccctgcatctggcgatcgtagcttttgcgtcgcaatggacgcagcatgcgcagaggggggctgggctaaatgttcctgcagacatagccgccgatgagaggtccatccggaggaacgcctggaatgaagcacgccatgccttgcagcacacgacagggattccatcattccgggttatatttgcgaatatcatcttttctctcacgcagagtgtgctggatgatgatgagcagcacggtatgggtgcacgtctagacaagctactcgaaaatgacggtgcgcccgtgttcctggaaaccgcgaaccgtcagctttatacattccgacataagtttgcacgaatgcaacgccgcggtaaggctttcaacaggctcccaggaggatctgtcgcatcgacattcgccggtattttcgagacaccgacgccgtcgtctgaaagcccacagcttgacccggttgtggccagtgaggagcatcgcagtacattaagccttatgttctggctagggatcatgttcgatacactaagcgctgcaatgtaccagcgaccactcgtggtgtcagatgaggatagccagatatcatcggcatctccaccaaggcgcggcgctgaaacgccgatcaacctagactgctgggagcccccgagacaggtcccgagcaatcaagaaaagagcgacgtatggggcgacctcttcctccgcacctcggactctctcccagatcacgaatcccacacacaaatctctcagccagcggctcgatggccctgcacctacgaacaggccgccgccgcactctcctctgcaacgcccgtcaaagtcctcctctaccgccgcgtcacgcagctccaaaccctcctctatcgcggcgcgagccctgcccgccttgaagcggccatccagagaacgctctacgtttataatcactggacagcgaagtaccaaccatttatgcaggactgcgttgctaaccacgagctgctcccttcgcgcatccagtcttggtacgtcattctagacggtcactggcatctagccgcgatgttgctagcggacgttttggagagcatcgaccgcgattcgtactctgatatcaaccacatcgaccttgtaacaaagctaaggctcgataatgcactagcagttagtgcccttgcgcgctcttcactccgaggccaggagctggacccaggcaaagcatctccgatgtatcgccatttccatgattctctgaccgaggtggcattcctggtagaaccgtggaccgtcgttcttattcactcgtttgccaaagctgcgtatatcttgctggactgtttagacctggacggccaaggaaatgcactagcgggctacctgcagctgcggcaaaattgcaactactgcattcgggcgctgcaatttctgggcaggaagtcggatatggcggcgctggttgcgaaggatttagagagaggtttgaatgggaaagttgacagctttttgtagaggcctattacttttactgttactaacgcatttcccatcctccaaaaatgcttttttaggcgtcagttggactatgctagcatcctctagagtcgacctgcaggcgttcaaacatttggcaataaagtttcttaagattgaatcctgttgccggtcttgcgatgattatcatataatttctgttgaattacgttaagcatgtaataattaacatgtaatgcatgacgttatttatgagatgggtttttatgattagagtcccgcaattatacatttaatacgcgatagaaaacaaaatatagcgcgcaaactaggataaattatcgcgcgcggtgtcatctatgttactagatcgggaattgccatgctttatgcgggatagttccgacctaggattggatgcatgcggaaccgcacgagggcggggcggaaattgacacaccactcctctccacgcaccgttcaagaggtacgcgtatagagccgtatagagcagagacggagcactttctggtactgtccgcacgggatgtccgcacggagagccacaaacgagcgggaccccgtacgtgctctcctaccccaggatcgcatccccgcatagctgaacatctatataactgcacgactctagaggatccccggcgcccaagtttgtacaaaaaagttgaacgagaaacgtaaaatgatataaatatcaatatattaaattagattttgcataaaaaacagactacataatactgtaaaacacaacatatgcagtcactatgaatcaactacttagatggtattagtgacctgtagactcactataggggaattgtgagcggataacaattcccctctagaaataattttgtttaactttaagaaggagatataatgcagtttaaggtttacacctataaaagagagagccgttatcgtctgtttgtggatgtacagagtgatattattgacacgcccggtcgacggatggtgatccccctggccagtgcacgtctgctgtcagataaagtcccccgtgaactttacccggtggtgcatatcggggatgaaagctggcgcatgatgaccaccgatatggccagtgtgccggtctccgttatcggggaagaagtggctgatctcagccaccgcgaaaatgacatcaaaaacgccattaacctgatgttctggggaatataaaaaaaacccctcaagacccgtttagaggccccaaggggttatgctatacaggtcactaataccatctaagtagttgattcatagtgactgcatatgttgtgttttacagtattatgtagtctgttttttatgcaaaatctaatttaatatattgatatttatatcattttacgtttctcgttcaactttcttgtacaaagtggtttaaactctagagtccgcaaaaatcaccagtctctctctacaaatctatctctctctatttttctccagaataatgtgtgagtagttcccagataagggaattagggttcttatagggtttcgctcatgtgttgagcatataagaaacccttagtatgtatttgtatttgtaaaatacttctatcaataaaatttctaattcctaaaaccaaaatccagtgaccgaattcggtactttaattaatgacaggatatattggcgggtaaactaagtcgctgtatgtgtttgtttgcgatcgcatgtgagcaaaaggccagcaaaaggccaggaaccgtaaaaaggccgcgttgctggcgtttttccataggctccgcccccctgacgagcatcacaaaaatcgacgctcaagtcagaggtggcgaaacccgacaggactataaagataccaggcgtttccccctggaagctccctcgtgcgctctcctgttccgaccctgccgcttaccggatacctgtccgcctttctcccttcgggaagcgtggcgctttctcatagctcacgctgtaggtatctcagttcggtgtaggtcgttcgctccaagctgggctgtgtgcacgaaccccccgttcagcccgaccgctgcgccttatccggtaactatcgtcttgagtccaacccggtaagacacgacttatcgccactggcagcagccactggtaacaggattagcagagcgaggtatgtaggcggtgctacagagttcttgaagtggtggcctaactacggctacactagaagaacagtatttggtatctgcgctctgctgaagccagttaccttcggaagaagagttggtagctcttgatccggcaaacaaaccaccgctggtagcggtggtttttttgtttgcaagcagcagattacgcgcagaaaaaaaggatctcaagaagatcctttgatcttttctacggggtctgacgctcagtggaacgaaaactcacgttaagggattttggtcatgagattatcaaaaaggatcttcacctagatccttttaaattaaaaatgaagttttaaatcaatctaaagtatatatgtgtaacattggtctagtgaatcgatttaccaatgcttaatcagtgaggcacctatctcagcgatctgtctatttcgttcatccatagttgcctgactccccgtcgtgtagataactacgatacgggagggcttaccatctggccccagtgctgcaatgataccgcgagacccacgctcaccggctccagatttatcagcaataaaccagccagccggaagggccgagcgcagaagtggtcctgcaactttatccgcctccatccagtctattaattgttgccgggaagctagagtaagtagttcgccagttaatagtttgcgcaacgttgttgccattgcagcaggcatcgtggtgtcacgctcgtcgtttggtatggcttcattcagctccggttcccaacgatcaaggcgagttacatgatcccccatgttgtgcaaaaaagcggttagctccttcggtcctcctatcgttgtcagaagtaagttggccgcagtgttatcactcatggttatggcagcactgcataattctcttactgtcatgccatccgtaagatgcttttctgtgactggtgaatactcaaccaagtcattctgagaatagtgtatgcggcgaccgagttgctcttgcccggcgtcaacacgggataataccgcgccacatagcagaactttaaaagtgctcatcattggaaaacgttcttcggggcgaaaactctcaaggatcttaccgctgttgagatccagttcgatgtaacccactcgtgcacccaactgatcttcagcatcttttactttcaccagcgtttctgggtgagcaaaaacaggaaggcaaaatgccgcaaaaaagggaataagggcgacacggaaatgttgaatactcatgctagcgttggaatttaatcgcggccttgagcaagacgtttcccgttgaatatggctcataacaccccttgtattactgtttatgtaagcagacagttttattgttcatgatgatatatttttatcttgtgcaatgtaacatcagagattttgagacacaacgtggctttgttgaataaatcgaacttttgctgagttgaaggatcagatcacgcatcttcccgacaacgcagaccgttccgtggcaaagcaaaagttcaaaatcaccaactggtccacctacaacaaagctctcatcaaccgtggctccctcactttctggctggatgatggggcgattcaggcgatccccatccaacagcccgccgtcgagcgggcttttttatccccggaagcctgtggatagagggtagttatccacgtgaaaccgctaatgccccgcaaagccttgattcacggggctttccggcccgctccaaaaactatccacgtgaaatcgctaatcagggtacgtgaaatcgctaatcggagtacgtgaaatcgctaataaggtcacgtgaaatcgctaatcaaaaaggcacgtgagaacgctaatagccctttcagatcaacagcttgcaaacacccctcgctccggcaagtagttacagcaagtagtatgttcaattagcttttcaattatgaatatatatatcaattattggtcgcccttggcttgtggacaatgcgctacgcgcaccggctccgcccgtggacaaccgcaagcggttgcccaccgtcgagcgccagcgcctttgcccacaacccggcggccggccgcaacagatcgttttataaatttttttttttgaaaaagaaaaagcccgaaaggcggcaacctctcgggcttctggatttccgatccccggaattag

1. **Sequences of markers/tags and the enhancer**
   1. Sequences of markers/tags
      1. CFP

atggtgagcaagggcgaggagctgttcaccggggtggtgcccatcctggtcgagctggacggcgacgtaaacggccacaggttcagcgtgtccggcgagggcgagggcgatgccacctacggcaagctgaccctgaagttcatctgcaccaccggcaagctgcccgtgccctggcccaccctcgtgaccaccctgacctggggcgtgcagtgcttcagccgctaccccgaccacatgaagcagcacgacttcttcaagtccgccatgcccgaaggctacgtccaggagcgtaccatcttcttcaaggacgacggcaactacaagacccgcgccgaggtgaagttcgagggcgacaccctggtgaaccgcatcgagctgaagggcatcgacttcaaggaggacggcaacatcctggggcacaagctggagtacaactacatcagccacaacgtctatatcaccgccgacaagcagaagaacggcatcaaggcccacttcaagatccgccacaacatcgaggacggcagcgtgcagctcgccgaccactaccagcagaacacccccatcggcgacggccccgtgctgctgcccgacaaccactacctgagcacccagtccgccctgagcaaagaccccaacgagaagcgcgatcacatggtcctgctggagttcgtgaccgccgccgggatcactctcggcatggacgagctgtacaag

- - 1. GFP

ATGGTGAGCAAGGGCGAGGAGCTGTTCACCGGGGTGGTGCCCATCCTGGTCGAGCTGGACGGCGACGTAAACGGCCACAAGTTCAGCGTGTCCGGCGAGGGCGAGGGCGATGCCACCTACGGCAAGCTGACCCTGAAGTTCATCTGCACCACCGGCAAGCTGCCCGTGCCCTGGCCCACCCTCGTGACCACCCTGACCTACGGCGTGCAGTGCTTCAGCCGCTACCCCGACCACATGAAGCAGCACGACTTCTTCAAGTCCGCCATGCCCGAAGGCTACGTCCAGGAGCGCACCATCTTCTTCAAGGACGACGGCAACTACAAGACCCGCGCCGAGGTGAAGTTCGAGGGCGACACCCTGGTGAACCGCATCGAGCTGAAGGGCATCGACTTCAAGGAGGACGGCAACATCCTGGGGCACAAGCTGGAGTACAACTACAACAGCCACAACGTCTATATCATGGCCGACAAGCAGAAGAACGGCATCAAGGTGAACTTCAAGATCCGCCACAACATCGAGGACGGCAGCGTGCAGCTCGCCGACCACTACCAGCAGAACACCCCCATCGGCGACGGCCCCGTGCTGCTGCCCGACAACCACTACCTGAGCACCCAGTCCGCCCTGAGCAAAGACCCCAACGAGAAGCGCGATCACATGGTCCTGCTGGAGTTCGTGACCGCCGCCGGGATCACTCTCGGCATGGACGAGCTGTACAAGGACGAGCTG

- - 1. YFP

atggtgagcaagggcgaggagctgttcaccggggtggtgcccatcctggtcgagctggacggcgacgtaaacggccacaagttcagcgtgtccggcgagggcgagggcgatgccacctacggcaagctgaccctgaagttcatctgcaccaccggcaagctgcccgtgccctggcccaccctcgtgaccaccttcggctacggcgtgcagtgcttcgcccgctaccccgaccacatgaagcagcacgacttcttcaagtccgccatgcccgaaggctacgtccaggagcgcaccatcttcttcaaggacgacggcaactacaagacccgcgccgaggtgaagttcgagggcgacaccctggtgaaccgcatcgagctgaagggcatcgacttcaaggaggacggcaacatcctggggcacaagctggagtacaactacaacagccacaacgtctatatcatggccgacaagcagaagaacggcatcaaggtgaacttcaagatccgccacaacatcgaggacggcagcgtgcagctcgccgaccactaccagcagaacacccccatcggcgacggccccgtgctgctgcccgacaaccactacctgagctaccagtccgccctgagcaaagaccccaacgagaagcgcgatcacatggtcctgctggagttcgtgaccgccgccgggatcactctcggcatggacgagctgtacaag

- - 1. YFPc

gacaagcagaagaacggcatcaaggtgaacttcaagatccgccacaacatcgaggacggcagcgtgcagctcgccgaccactaccagcagaacacccccatcggcgacggccccgtgctgctgcccgacaaccactacctgagctaccagtccgccctgagcaaagaccccaacgagaagcgcgatcacatggtcctgctggagttcgtgaccgccgccgggatcactctcggcatggacgagctgtacaag

- - 1. YFPn

atggtgagcaagggcgaggagctgttcaccggggtggtgcccatcctggtcgagctggacggcgacgtaaacggccacaagttcagcgtgtccggcgagggcgagggcgatgccacctacggcaagctgaccctgaagttcatctgcaccaccggcaagctgcccgtgccctggcccaccctcgtgaccaccttcggctacggcgtgcagtgcttcgcccgctaccccgaccacatgaagcagcacgacttcttcaagtccgccatgcccgaaggctacgtccaggagcgcaccatcttcttcaaggacgacggcaactacaagacccgcgccgaggtgaagttcgagggcgacaccctggtgaaccgcatcgagctgaagggcatcgacttcaaggaggacggcaacatcctggggcacaagctggagtacaactacaacagccacaacgtctatatcatggcc

- - 1. mRFP

atggcctcctccgaggacgtcatcaaggagttcatgcgcttcaaggtgcgcatggagggctccgtgaacggccacgagttcgagatcgagggcgagggcgagggccgcccctacgagggcacccagaccgccaagctgaaggtgaccaagggcggccccctgcccttcgcctgggacatcctgtcccctcagttccagtacggctccaaggcctacgtgaagcaccccgccgacatccccgactacttgaagctgtccttccccgagggcttcaagtgggagcgcgtgatgaacttcgaggacggcggcgtggtgaccgtgacccaggactcctccctgcaggacggcgagttcatctacaaggtgaagctgcgcggcaccaacttcccctccgacggccccgtaatgcagaagaagaccatgggctgggaggcctccaccgagcggatgtaccccgaggacggcgccctgaagggcgagatcaagatgaggctgaagctgaaggacggcggccactacgacgccgaggtcaagaccacctacatggccaagaagcccgtgcagctgcccggcgcctacaagaccgacatcaagctggacatcacctcccacaacgaggactacaccatcgtggaacagtacgagcgcgccgagggccgccactccaccggcgcctaa

- - 1. GST

ATGTCCCCTATACTAGGTTATTGGAAAATTAAGGGCCTTGTGCAACCCACTCGACTTCTTTTGGAATATCTTGAAGAAAAATATGAAGAGCATTTGTATGAGCGCGATGAAGGTGATAAATGGCGAAACAAAAAGTTTGAATTGGGTTTGGAGTTTCCCAATCTTCCTTATTATATTGATGGTGATGTTAAATTAACACAGTCTATGGCCATCATACGTTATATAGCTGACAAGCACAACATGTTGGGTGGTTGTCCAAAAGAGCGTGCAGAGATTTCAATGCTTGAAGGAGCGGTTTTGGATATTAGATACGGTGTTTCGAGAATTGCATATAGTAAAGACTTTGAAACTCTCAAAGTTGATTTTCTTAGCAAGCTACCTGAAATGCTGAAAATGTTCGAAGATCGTTTATGTCATAAAACATATTTAAATGGTGATCATGTAACCCATCCTGACTTCATGTTGTATGACGCTCTTGATGTTGTTTTATACATGGACCCAATGTGCCTGGATGCGTTCCCAAAATTAGTTTGTTTTAAAAAACGTATTGAAGCTATCCCACAAATTGATAAGTACTTGAAATCCAGCAAGTATATAGCATGGCCTTTGCAGGGCTGGCAAGCCACGTTTGGTGGTGGCGACCATCCTCCAAAATCGGATCTGGTTCCGCGT

- - 1. GUS

ATGTTACGTCCTGTAGAAACCCCAACCCGTGAAATCAAAAAACTCGACGGCCTGTGGGCATTCAGTCTGGATCGCGAAAACTGTGGAATTGATCAGCGTTGGTGGGAAAGCGCGTTACAAGAAAGCCGGGCAATTGCTGTGCCAGGCAGTTTTAACGATCAGTTCGCCGATGCAGATATTCGTAATTATGCGGGCAACGTCTGGTATCAGCGCGAAGTCTTTATACCGAAAGGTTGGGCAGGCCAGCGTATCGTGCTGCGTTTCGATGCGGTCACTCATTACGGCAAAGTGTGGGTCAATAATCAGGAAGTGATGGAGCATCAGGGCGGCTATACGCCATTTGAAGCCGATGTCACGCCGTATGTTATTGCCGGGAAAAGTGTACGTATCACCGTTTGTGTGAACAACGAACTGAACTGGCAGACTATCCCGCCGGGAATGGTGATTACCGACGAAAACGGCAAGAAAAAGCAGTCTTACTTCCATGATTTCTTTAACTATGCCGGAATCCATCGCAGCGTAATGCTCTACACCACGCCGAACACCTGGGTGGACGATATCACCGTGGTGACGCATGTCGCGCAAGACTGTAACCACGCGTCTGTTGACTGGCAGGTGGTGGCCAATGGTGATGTCAGCGTTGAACTGCGTGATGCGGATCAACAGGTGGTTGCAACTGGACAAGGCACTAGCGGGACTTTGCAAGTGGTGAATCCGCACCTCTGGCAACCGGGTGAAGGTTATCTCTATGAACTGTGCGTCACAGCCAAAAGCCAGACAGAGTGTGATATCTACCCGCTTCGCGTCGGCATCCGGTCAGTGGCAGTGAAGGGCCAACAGTTCCTGATTAACCACAAACCGTTCTACTTTACTGGCTTTGGTCGTCATGAAGATGCGGACTTACGTGGCAAAGGATTCGATAACGTGCTGATGGTGCACGACCACGCATTAATGGACTGGATTGGGGCCAACTCCTACCGTACCTCGCATTACCCTTACGCTGAAGAGATGCTCGACTGGGCAGATGAACATGGCATCGTGGTGATTGATGAAACTGCTGCTGTCGGCTTTAACCTCTCTTTAGGCATTGGTTTCGAAGCGGGCAACAAGCCGAAAGAACTGTACAGCGAAGAGGCAGTCAACGGGGAAACTCAGCAAGCGCACTTACAGGCGATTAAAGAGCTGATAGCGCGTGACAAAAACCACCCAAGCGTGGTGATGTGGAGTATTGCCAACGAACCGGATACCCGTCCGCAAGTGCACGGGAATATTTCGCCACTGGCGGAAGCAACGCGTAAACTCGACCCGACGCGTCCGATCACCTGCGTCAATGTAATGTTCTGCGACGCTCACACCGATACCATCAGCGATCTCTTTGATGTGCTGTGCCTGAACCGTTATTACGGATGGTATGTCCAAAGCGGCGATTTGGAAACGGCAGAGAAGGTACTGGAAAAAGAACTTCTGGCCTGGCAGGAGAAACTGCATCAGCCGATTATCATCACCGAATACGGCGTGGATACGTTAGCCGGGCTGCACTCAATGTACACCGACATGTGGAGTGAAGAGTATCAGTGTGCATGGCTGGATATGTATCACCGCGTCTTTGATCGCGTCAGCGCCGTCGTCGGTGAACAGGTATGGAATTTCGCCGATTTTGCGACCTCGCAAGGCATATTGCGCGTTGGCGGTAACAAGAAAGGGATCTTCACTCGCGACCGCAAACCGAAGTCGGCGGCTTTTCTGCTGCAAAAACGCTGGACTGGCATGAACTTCGGTGAAAAACCGCAGCAGGGAGGCAAACAATGA

- - 1. LUC

ATGGAAGACGCCAAAAACATAAAGAAAGGCCCGGCGCCATTCTATCCGCTGGAAGATGGAACCGCTGGAGAGCAACTGCATAAGGCTATGAAGAGATACGCCCTGGTTCCTGGAACAATTGCTTTTACAGATGCACATATCGAGGTGGACATCACTTACGCTGAGTACTTCGAAATGTCCGTTCGGTTGGCAGAAGCTATGAAACGATATGGGCTGAATACAAATCACAGAATCGTCGTATGCAGTGAAAACTCTCTTCAATTCTTTATGCCGGTGTTGGGCGCGTTATTTATCGGAGTTGCAGTTGCGCCCGCGAACGACATTTATAATGAACGTGAATTGCTCAACAGTATGGGCATTTCGCAGCCTACCGTGGTGTTCGTTTCCAAAAAGGGGTTGCAAAAAATTTTGAACGTGCAAAAAAAGCTCCCAATCATCCAAAAAATTATTATCATGGATTCTAAAACGGATTACCAGGGATTTCAGTCGATGTACACGTTCGTCACATCTCATCTACCTCCCGGTTTTAATGAATACGATTTTGTGCCAGAGTCCTTCGATAGGGACAAGACAATTGCACTGATCATGAACTCCTCTGGATCTACTGGTCTGCCTAAAGGTGTCGCTCTGCCTCATAGAACTGCCTGCGTGAGATTCTCGCATGCCAGAGATCCTATTTTTGGCAATCAAATCATTCCGGATACTGCGATTTTAAGTGTTGTTCCATTCCATCACGGTTTTGGAATGTTTACTACACTCGGATATTTGATATGTGGATTTCGAGTCGTCTTAATGTATAGATTTGAAGAAGAGCTGTTTCTGAGGAGCCTTCAGGATTACAAGATTCAAAGTGCGCTGCTGGTGCCAACCCTATTCTCCTTCTTCGCCAAAAGCACTCTGATTGACAAATACGATTTATCTAATTTACACGAAATTGCTTCTGGTGGCGCTCCCCTCTCTAAGGAAGTCGGGGAAGCGGTTGCCAAGAGGTTCCATCTGCCAGGTATCAGGCAAGGATATGGGCTCACTGAGACTACATCAGCTATTCTGATTACACCCGAGGGGGATGATAAACCGGGCGCGGTCGGTAAAGTTGTTCCATTTTTTGAAGCGAAGGTTGTGGATCTGGATACCGGGAAAACGCTGGGCGTTAATCAAAGAGGCGAACTGTGTGTGAGAGGTCCTATGATTATGTCCGGTTATGTAAACAATCCGGAAGCGACCAACGCCTTGATTGACAAGGATGGATGGCTACATTCTGGAGACATAGCTTACTGGGACGAAGACGAACACTTCTTCATCGTTGACCGCCTGAAGTCTCTGATTAAGTACAAAGGCTATCAGGTGGCTCCCGCTGAATTGGAATCCATCTTGCTCCAACACCCCAACATCTTCGACGCAGGTGTCGCAGGTCTTCCCGACGATGACGCCGGTGAACTTCCCGCCGCCGTTGTTGTTTTGGAGCACGGAAAGACGATGACGGAAAAAGAGATCGTGGATTACGTCGCCAGTCAAGTAACAACCGCGAAAAAGTTGCGCGGAGGAGTTGTGTTTGTGGACGAAGTACCGAAAGGTCTTACCGGAAAACTCGACGCAAGAAAAATCAGAGAGATCCTCATAAAGGCCAAGAAGGGCGGAAAGATCGCCGTGTAA

- - 1. MYC

GAGCAAAAGCTTATCTCTGAGGAAGACTTG

- - 1. 3XMYC

GAGCAAAAGCTTATCTCTGAGGAAGACTTGggcgccGAACAGAAATTGATTTCAGAGGAAGACCTTcaattgGAACAAAAGCTCATCTCTGAAGAAGACCTC

- - 1. HA

TACCCATACGATGTTCCTGACTACGCT

- - 1. 3XHA

TACCCATACGATGTTCCTGACTACGCTggcgccTATCCCTATGACGTCCCGGACTATGCAcaattgTATCCTTACGATGTGCCAGATTACGCC

- - 1. FLAG

GACTACAAGGATGACGATGACAAG

- - 1. 3xFLAG

GACTACAAGGATGACGATGACAAGggcgccGATTATAAAGATGACGATGACAAGcaattgGACTATAAGGACGATGACGATAAA

- - 1. StrepII

TGGAGCCACCCGCAGTTCGAAAAG

- - 1. NLS

CGACCCCCAAAGAAGAAGCGTAAGGTT

- - 1. NES

AACGAGCTTGCTCTTAAGTTGGCTGGACTTGATATTAACAAGACTGGAGGA

- 1. Sequences of the enhancer

gatccccaacatggtggagcacgacactctcgtctactccaagaatatcaaagatacagtctcagaagaccagagggctattgagacttttcaacaaagggtaatatcgggaaacctcctcggattccattgcccagctatctgtcacttcatcgaaaggacagtagaaaaggaagatggcttctacaaatgccatcattgcgataaaggaaaggctatcgttcaagatgcctctaccgacagtggtcccaaagatggacccccacccacgaggaacatcgtggaaaaagaagacgttccaaccacgtcttcaaagcaagtggattgatgtgatattca

- 1. Sequences of resistant genes
     1. Specinomycin

atgagggaagcggtgatcgccgaagtatcgactcaactatcagaggtagttggcgtcatcgagcgccatctcgaaccgacgttgctggccgtacatttgtacggctccgcagtggatggcggcctgaagccacacagtgatattgatttgctggttacggtgaccgtaaggcttgatgaaacaacgcggcgagctttgatcaacgaccttttggaaacttcggcttcccctggagagagcgagattctccgcgctgtagaagtcaccattgttgtgcacgacgacatcattccgtggcgttatccagctaagcgcgaactgcaatttggagaatggcagcgcaatgacattcttgcaggtatcttcgagccagccaccatcgacattgatctggctatcttgctgacaaaagcaagagaacatagcgttgccttggtaggtccagcggcggaggaactctttgatccggttcctgaacaggatctatttgaggcgctaaatgaaaccttaacgctatggaactcgccgcccgactgggctggcgatgagcgaaatgtagtgcttacgttgtcccgcatttggtacagcgcagtaaccggcaaaatcgcgccgaaggatgtcgctgccgactgggcaatggagcgcctgccggcccagtatcagcccgtcatacttgaagctaggcaggcttatcttggacaagaagatcgcttggcctcgcgcgcagatcagttggaagaatttgttcactacgtgaaaggcgagatcaccaaggtagtcggcaaataa

- - 1. Kanamycin

atgattgaacaagatggattgcacgcaggttctccggccgcttgggtggagaggctattcggctatgactgggcacaacagacaatcggctgctctgatgccgccgtgttccggctgtcagcgcaggggcgcccggttctttttgtcaagaccgacctgtccggtgccctgaatgaactgcaggacgaggcagcgcggctatcgtggctggccacgacgggcgttccttgcgcagctgtgctcgacgttgtcactgaagcgggaagggactggctgctattgggcgaagtgccggggcaggatctcctgtcatctcaccttgctcctgccgagaaagtatccatcatggctgatgcaatgcggcggctgcatacgcttgatccggctacctgcccattcgaccaccaagcgaaacatcgcatcgagcgagcacgtactcggatggaagccggtcttgtcgatcaggatgatctggacgaagagcatcaggggctcgcgccagccgaactgttcgccaggctcaaggcgcgcatgcccgacggcgatgatctcgtcgtgacccatggcgatgcctgcttgccgaatatcatggtggaaaatggccgcttttctggattcatcgactgtggccggctgggtgtggcggaccgctatcaggacatagcgttggctacccgtgatattgctgaagagcttggcggcgaatgggctgaccgcttcctcgtgctttacggtatcgccgctcccgattcgcagcgcatcgccttctatcgccttcttgacgagttcttctga

- - 1. Ampicilin

ATGAGTATTCAACATTTCCGTGTCGCCCTTATTCCCTTTTTTGCGGCATTTTGCCTTCCTGTTTTTGCTCACCCAGAAACGCTGGTGAAAGTAAAAGATGCTGAAGATCAGTTGGGTGCACGAGTGGGTTACATCGAACTGGATCTCAACAGCGGTAAGATCCTTGAGAGTTTTCGCCCCGAAGAACGTTTTCCAATGATGAGCACTTTTAAAGTTCTGCTATGTGGCGCGGTATTATCCCGTGTTGACGCCGGGCAAGAGCAACTCGGTCGCCGCATACACTATTCTCAGAATGACTTGGTTGAGTAtTCACCAGTCACAGAAAAGCATCTTACGGATGGCATGACAGTAAGAGAATTATGCAGTGCTGCCATAACCATGAGTGATAACACTGCGGCCAACTTACTTCTGACAACGATaGGAGGACCGAAGGAGCTAACCGCTTTTTTGCACAACATGGGGGATCATGTAACTCGCCTTGATCGTTGGGAACCGGAGCTGAATGAAGCCATACCAAACGACGAGCGTGACACCACGATGCCTGCtGCAATGGCAACAACGTTGCGCAAACTATTAACTGGCGAACTACTTACTCTAGCTTCCCGGCAACAATTAATAGACTGGATGGAGGCGGATAAAGTTGCAGGACCACTTCTGCGCTCGGCCCTTCCGGCTGGCTGGTTTATTGCTGATAAATCTGGAGCCGGTGAGCGTGGGTCTCGCGGTATCATTGCAGCACTGGGGCCAGATGGTAAGCCCTCCCGTATCGTAGTTATCTACACGACGGGGAGTCAGGCAACTATGGATGAACGAAATAGACAGATCGCTGAGATAGGTGCCTCACTGATTAAGCATTGGTAA

- - 1. Glufosinate

ATGAGCCCAGAACGACGCCCGGCCGACATCCGCCGTGCCACCGAGGCGGACATGCCGGCGGTCTGCACCATCGTCAACCACTACATCGAGACAAGCACGGTCAACTTCCGTACCGAGCCGCAGGAACCGCAGGAGTGGACGGACGACCTCGTCCGTCTGCGGGAGCGCTATCCCTGGCTCGTCGCCGAGGTGGACGGCGAGGTCGCCGGCATCGCCTACGCGGGaCCCTGGAAGGCACGCAACGCCTACGACTGGACGGCCGAGTCaACCGTGTACGTCTCCCCCCGCCACCAGCGGACGGGACTGGGCTCCACGCTCTACACCCACCTGCTGAAGTCCCTGGAGGCACAGGGCTTCAAGAGCGTGGTCGCTGTCATCGGGCTGCCCAACGACCCGAGCGTGCGaATGCACGAGGCGCTCGGATATGCCCCCCGCGGaATGCTGCGGGCGGCCGGCTTCAAGCACGGGAACTGGCATGACGTGGGTTTCTGGCAGCTGGACTTCAGCCTGCCGGTGCCGCCCCGTCCGGTCCTGCCCGTCACCGAAATCTGA

- - 1. Chloramphenicol

atgcagtttaaggtttacacctataaaagagagagccgttatcgtctgtttgtggatgtacagagtgatattattgacacgcccgggcgacggatggtgatccccctggccagtgcacgtctgctgtcagataaagtcccccgtgaactttacccggtggtgcatatcggggatgaaagctggcgcatgatgaccaccgatatggccagtgtgccggtctccgttatcggggaagaagtggctgatctcagccaccgcgaaaatgacatcaaaaacgccattaacctgatgttctggggaatataagtttaaacgcgctagcttaattaa

1. **Sequence of recombination sites**
   1. *att*L1

CAAATAATGATTTTATTTTGACTGATAGTGACCTGTTCGTTGCAACACATTGATGAGCAATGCTTTTTTATAATGCCAACTTTGTACAAAAAAGCAGGCTTT

- 1. *att*L2

ACCCAGCTTTCTTGTACAAAGTTGGCATTATAAGAAAGCATTGCTTATCAATTTGTTGCAACGAACAGGTCACTATCAGTCAAAATAAAATCATTATTTGC

- 1. *att*L3

aaataatgattttattttgactgatagtgacctgttcgttgcaacaaattgataagcaatgcttttttataatgccaactttgtataataaagttg

- 1. *att*L4

caacttttctatacaaagttggcattataaaaaagcattgcttatcaatttgttgcaacgaacaggtcactatcagtcaaaataaaatcattattt

- 1. *att*R1

acaagtttgtacaaaaaagctgaacgagaaacgtaaaatgatataaatatcaatatattaaattagattttgcataaaaaacagactacataatactgtaaaacacaacatatccagtcactatg

- 1. *att*R2

Catagtgactggatatgttgtgttttacagtattatgtagtctgttttttatgcaaaatctaatttaatatattgatatttatatcattttacgtttctcgttcagctttcttgtacaaagtggt

- 1. *att*R3

ccatagtgactggatatgttgtgttttacagtattatgtagtctgttttttatgcaaaatctaatttaatatattgatatttatatcattttacgtttctcgttcaacttttctatacaaagttg

- 1. *att*R4

aactatgtataataaagttgaacgagaaacgtaaaatgatataaatatcaatatattaaattagattttgcataaaaaacagactacataatactgtaaaacacaacatatccagtcactatgg
